# Unnatural spirocyclic oxindole alkaloids biosynthesis in *Uncaria guianensis*

**DOI:** 10.1038/s41598-019-47706-3

**Published:** 2019-08-05

**Authors:** Adriana A. Lopes, Bianca Chioca, Bruno Musquiari, Eduardo J. Crevelin, Suzelei de C. França, Maria Fatima das G. Fernandes da Silva, Ana Maria S. Pereira

**Affiliations:** 1 Unidade de Biotecnologia, Universidade de Ribeirão Preto (UNAERP), Av. Costábile Romano, 2201, 14096-900 Ribeirão Preto, SP Brazil; 2 Faculdade de Filosofia, Ciências e Letras de Ribeirão Preto, Universidade de São Paulo (USP), Av. do Café s/n, 14040-900 Ribeirão Preto, SP Brazil; 3 Centro de Ciências Exatas e de Tecnologia, Departamento de Química, Universidade Federal de São Carlos (UFSCar), Rod. Washington Luis s/n, 13565-905 São Carlos, SP Brazil

**Keywords:** Plant sciences, Biosynthesis

## Abstract

Spiro-oxindole scaffolds have been studied due to their promising therapeutic potential. In the Amazon rainforest there are two important *Uncaria* species known as “cat’s claw”, which biosynthesize spirocyclic oxindole alkaloids; *Uncaria tomentosa* (Willd. ex Schult.) DC. and *Uncaria guianensis* (Aublet) Gmell. We carried out a precursor-directed biosynthesis approach with *U. guianensis* and successfully obtained oxindole alkaloid analogues with molecular mass corresponding to the addition of a methyl or fluorine group on the oxindole ring using tryptamine analogue precursors. Two of these novel oxindole alkaloid analogues (**3b**-7-methyl-isomitraphylline and **3c**-6-fluoro-isomitraphylline) were isolated and characterized by NMR spectroscopy and ESI-QTOF-MS/MS. Having established a substrate feeding protocol for these plantlets, the biosynthetic route for mitraphylline (**1**), rhynchophylline (**2**), isomitraphylline (**3**) and isorhynchophylline (**4**) was also investigated using ^13^C-precursors (1-^13^C-D-glucose, 2-^13^C-tryptophan, 1-^13^C-DL-glyceraldehyde, and methyl-^13^C-D-methionine).

## Introduction

Natural products and their derivatives have been, and continue to be, a source of inspiration in the drug discovery domain. Currently, between 50–70% of all small molecule therapeutics used today are natural product inspired^1^. In general, modifications to the natural product structure to yield “new-to-nature compounds” often result in either improved biological or pharmacological activity. As an example, fludrocorticoid was the first fluorine drug identified as an active glucocorticoid, displaying 10-fold more potency than other halogenated cortisone derivatives^2^. Additionally, the fluorovinblastine analogue exhibits remarkable antitumor activity (IC_50_ = 300 pM) displaying 30-fold more potency than vinblastine (IC_50_ = 10 nM)^3^, and two fluorinated camptothecin analogues showed potent cytotoxicity against the multidrug-resistant KB-VIN cell line, and were more effective than irinotecan. Remarkably, more than 20% of the drugs currently available contain at least one fluorine atom^4^. For this reason, precursor-directed biosynthesis^5,6^ and metabolic engineering strategies^7–12^ have been explored in medicinal plants in order to generate novel fluorinated analogues. Precursor-directed biosynthesis procedures with *Catharanthus roseus* plants and tissue have already demonstrated that is it possible to produce unnatural monoterpene indole alkaloids^5,6^. The spirocyclic monoterpene oxindole alkaloids from *Uncaria*, which are biosynthetically related to the monoterpene indole alkaloids of *C. roseus*, have been investigated due to the therapeutic potential of the oxindole nucleus that is found in numerous natural products^13,14^. *Uncaria* (Rubiaceae) is a prolific producer of the bioactive spirocyclic oxindole alkaloids, and in the Brazilian Amazon two economically important *Uncaria* species are prevalent; *Uncaria tomentosa* (Willd. ex Schult.) DC. and *Uncaria guianensis* (Aublet) Gmell. These species are popularly known as “cat’s claw” and have attracted attention for their value in medicine, displaying a wide range of pharmacological activities, such as anticancer^15^, anti-inflammatory^16,17^ and immunomodulation effects^18^. In spite of many pharmacological studies, little work has been reported about the biosynthetic aspects of these compounds; only one ^14^C-feeding experiment has reported the elucidation of the oxindole alkaloid biosynthesis in *Mitragyna parvifolia* (Roxb.) Korth (Rubiaceae)^19^. Thus, here we develop a process for substrate feeding to plantlets of *U. guianensis*. We use this methodology first to perform a precursor-directed biosynthesis approach by feeding tryptamine analogs (**a**:5-methyl-tryptamine, **b**:7-methyl-tryptamine, and **c**:6-fluoro-tryptamine) to *U. guianensis* plantlets to obtain unnatural spirocyclic oxindole alkaloids. Two of the most abundant alkaloid analogues were isolated and subjected to 1D and 2D NMR and HRMS. Moreover, in our continuous efforts to unravel the biosynthetic details of the oxindole alkaloid biosynthetic pathway, we also applied this feeding strategy with ^13^C-precusors (1-^13^C-D-glucose, 2-^13^C-tryptophan, 1-^13^C-glyceraldehyde. and methyl-^13^C-D-methionine).

## Results

### Unnatural feeding experiments

**Figure 1:**
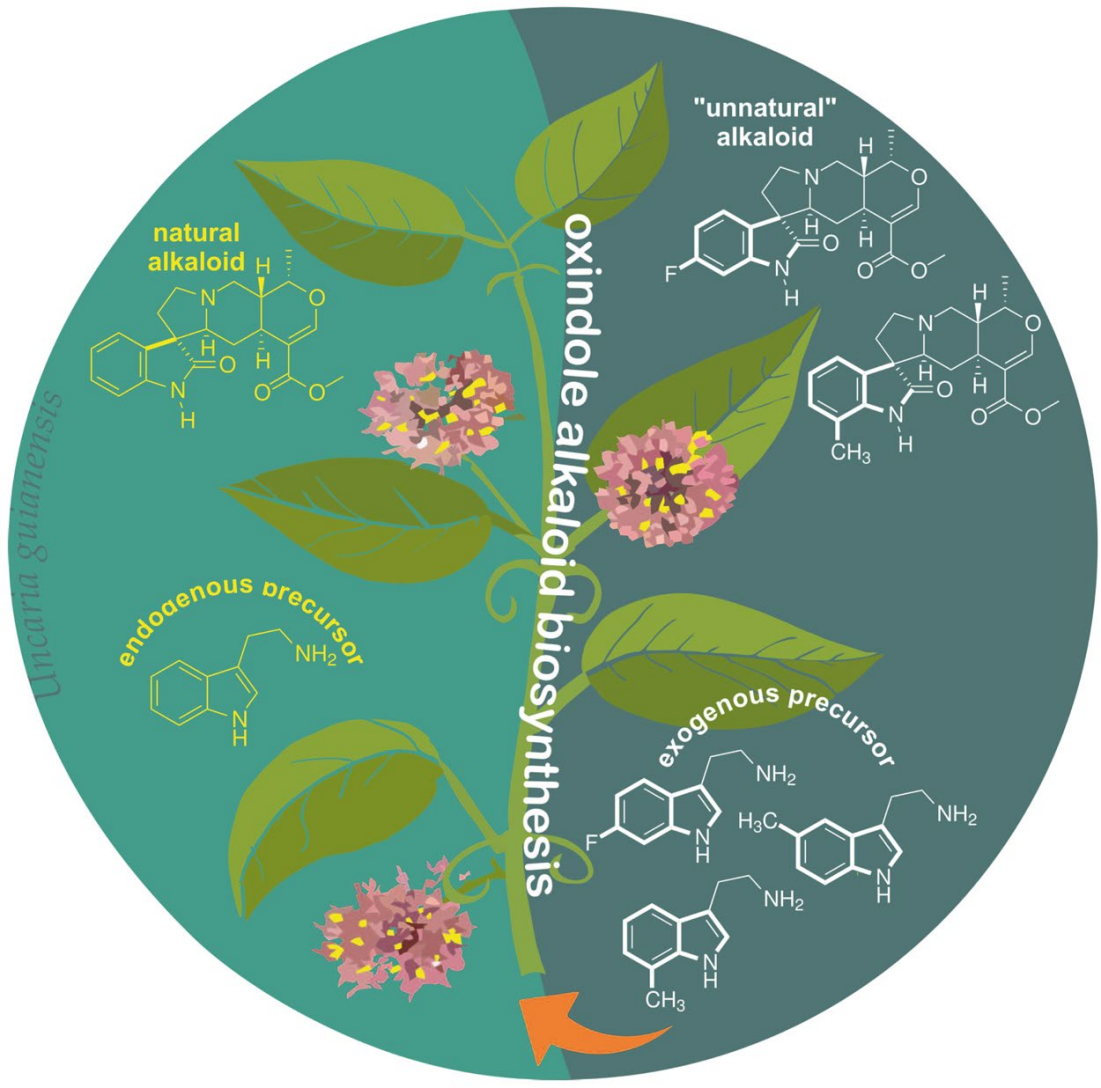
General strategies for oxindole alkaloid analogues design using precursor-directed biosynthesis in *U. guianensis* plantlets.

**Figure 2:**
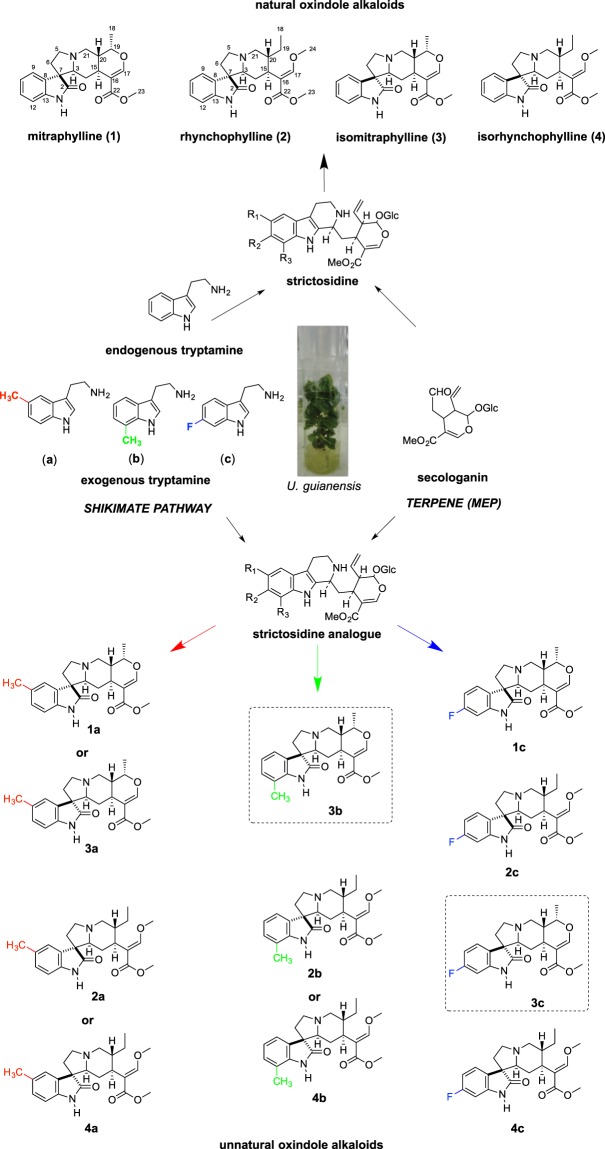
Natural and unnatural oxindole alkaloids biosynthesized by *U. guianensis* plantlets. Square (dashed lines) represent complete characterization of new-to-nature oxindole alkaloids.

**Figure 3:**
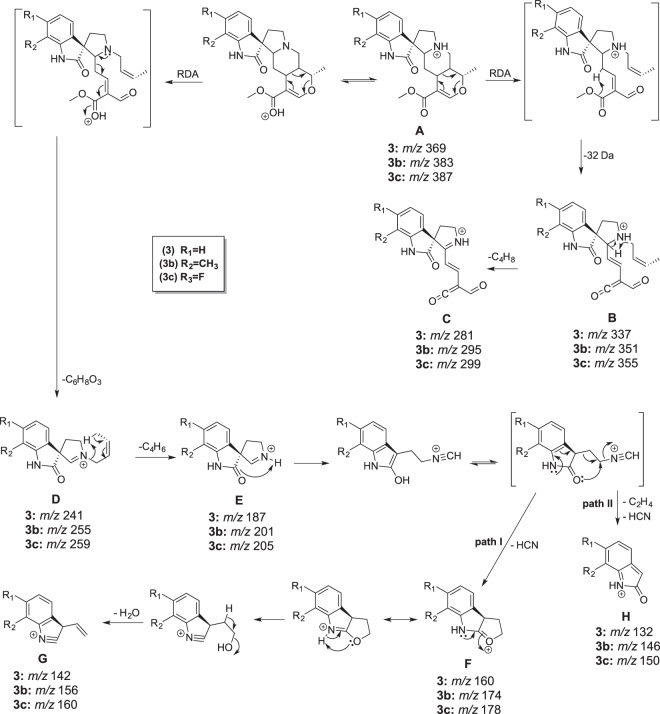
Formation of the main product ions of natural and unnatural oxindole alkaloids **3**, **3b** and **3c**.

Shoots of *U. guianensis* (2 months old) were transferred to sterile liquid media supplemented with the tryptamine analogs **a**-**c** (Fig. 1). A ethanolic extract from fresh shoots of *U. guianensis* was prepared after 30 days of culture with the tryptamine analogues and assessed by UPLC-DAD-MS. *U. guianensis* shoots produce natural pentacyclic oxindole alkaloids (POA) **1** and **3**, and tetracyclic oxindole alkaloids (TOA) **2** and **4** (Figs 2 and S1). After precursor-directed biosynthesis using **a**, in addition to the four natural alkaloids (**1**–**4**); **1** (*m/z* 369.1820 [M + H]^+^) and epimer **3** (*m/z* 369.1823 [M + H]^+^), **2** (*m/z* 385.2140 [M + H]^+^) and epimer **4** (*m/z* 385.2142 [M + H]^+^); just two additional peaks corresponding to either 5-methyl-mitraphylline (**1a**) or 5-methyl-isomitraphylline (**3a**) (*m/z* = 383.1982 [M + H^+^]) and either 5-methyl-rhynchophylline (**2a**) or 5-methyl-isorhynchophylline (**4a**) (*m/z* = 399.2291 [M + H^+^]) were observed. Similar results were obtained by incubation with **b** precursor, corresponding to either 7-methyl-mitraphylline (**1b**) or 7-methyl-isomitraphylline (**3b**) (*m/z* = 383.1985 [M + H^+^]) and either 7-methyl-rhynchophylline (**2b**) or 7-methyl-isorhynchophylline (**4b**) (*m/z* = 399.2295 [M + H^+^]). After incubation with **c**, the extracted-ion chromatograms showed the presence of four natural alkaloids (**1–4**), as well as four new compounds with masses corresponding to 6-fluoro-mitraphylline (**1c)** (*m/z* 387.1734 [M + H]^+^), 6-fluoro-isomitraphylline (**3c**) (m/z = 387.1730 [M + H^+^]), 6-fluoro-rhynchophylline (**2c**) (*m/z* 403.2025 [M + H]^+^) and 6-fluoro-isorhynchophylline (**4c**) (*m/z* = 403.2023 [M + H^+^]) (Figs S2–S6 and Table S1). Having established that *U. guianensis* could produce unnatural alkaloids, these precursor-directed biosynthesis approaches were performed on a large scale using **b-c** precursors. After 30 days of culture, ethanolic extracts were prepared and submitted to chromatographic procedures and semi-preparative HPLC. Compounds **3b** and **3c** were purified and characterized by 1D and 2D NMR (Figs S27–S36 and Table S2) and HRMS (Fig. S37 and Table S3). NMR data from alkaloid **3** showed five important cross signal correlations by HMQC; one related to double bond and four related to aromatic hydrogens (Fig. S20). The unnatural alkaloids **3b** and **3c** showed four cross signal correlations, showing that one position was substituted by either methyl or fluoride groups (Figs S30 and S35). Additionally, main product ions of natural and unnatural oxindole alkaloids **3**, **3b** and **3c** were confirmed by ESI-QTOF-MS/MS (Fig. 3). All NMR data and ESI-QTOF-MS/MS allowed us to establish the structure of these two novel oxindole alkaloids in comparation with our NMR data and literature data from natural oxindole alkaloids (**1** and **3**^20–22^; **2** and **4**^23^), and confirmed the capacity for efficient unnatural oxindole alkaloids production using precursor directed biosynthesis.

### Labeled feeding experiments

**Figure 4:**
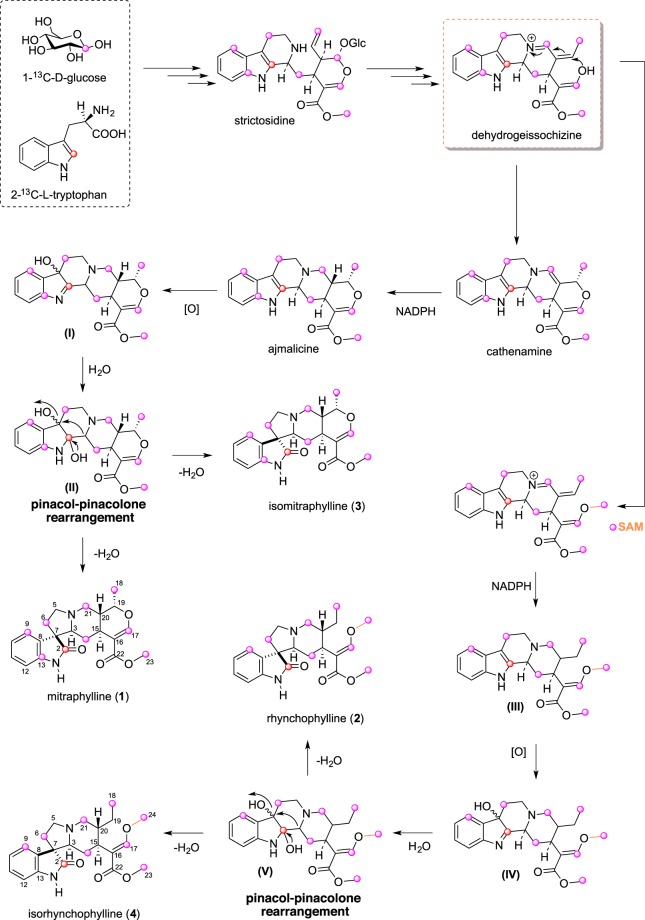
Labelling patterns obtained by biosynthetic precursor 1-^13^C-D-glucose and 2-^13^C-tryptophan into POA (**1** and **3**) and TOA (**2** and **4**).

Given the success of feeding of unnatural substrate analogues, and given that an understanding of the biosynthetic pathway can help to identify intermediates and enzymes involved in oxindole alkaloid biosynthesis, we used this platform to perform labelling studies of the alkaloids of *U. guianensis*. Segments of *U. guianensis* were inoculated in liquid medium supplemented with 1-^13^C-D-glucose (3% m/v), 2-^13^C-tryptophan (1 mM), 1-^13^C-DL-glyceraldehyde (1 mM) and methyl-^13^C-D-methionine (0.3% m/v). Additionally, a control group of plantlets that did not receive ^13^C-precursors was also prepared. After ten weeks of culture, an ethanolic extract from fresh shoots of *U. guianensis*, where the oxindole alkaloids **1**–**4** are the major constituents, was prepared. This ethanolic extract was fractioned by dowex^®^ resin and semi-preparative HPLC procedures to yield both labeled and non-labeled **1**, **3** and **4**. Incorporation patterns of labeled oxindole alkaloids were determined by quantitative ^13^C NMR (Figs S38–S45) by comparing the relative intensities of the labeled and non-labeled STL signals for **1**, **3** and **4** (Tables S4–S6). DL-glyceraldehyde and D-methionine ^13^C-precursors were not incorporated into oxindole biosynthesis by *U. guianensis* shoots. The ^13^C enrichment patterns of **1**, **3** and **4** after 1-^13^C-D-glucose metabolism indicated that the secologanin scaffold was predominantly formed by the MEP pathway, and oxindole moiety is derived from the shikimate pathway in both the POA (**1** and **3**) and TOA (**2** and **4**) framework. The ^13^C NMR spectrum of ^13^C-labeled **1** and **3** revealed enhancement of the signals at C-6, C-9, and C-13 derived from tryptamine metabolism; and C-14, C-17, C-18, and C-21, thus indicating incorporation of a label into the corresponding C-1 and C-5 of IPP derived from the MEP pathway (Fig. 4). The ^13^C NMR spectrum of labeled **4** presented a similar enrichment pattern for the corresponding carbon atoms at C-6, C-9, C-14, C-17, C-18, and C-21 from the TOA. Position C-23 from **1** and **3**, and positions C-23 and C-24 from **4** showed high enrichment, corresponding to SAM action. Isotopic labeling from the 2-^13^C-tryptophan experiment led to enrichment at positions C-2 of **1**, **3** and **4**, therefore indicating operation of the tryptamine into the oxindole alkaloids.

## Discussion

Our studies showed that precursor-directed biosynthesis strategies using analogue precursors produce successful unnatural oxindole alkaloids in *U. guianensis* shoots. Four unnatural fluoro-oxindole alkaloids were detected using 6-fluoro-tryptamine derivative (**c**); two TOA and two POA, while after incubation with methyl-precursors (**a**-**b**) only two unnatural methylated-oxindole alkaloids were detected; one TOA and one POA scaffold. These data suggest that the fluorinated **c** precursor is more acceptable to the biosynthetic enzymes in *U. guianensis* compared to **a**-**c** precursors as previously reported in *C. roseus*^7^. It is important to point out that *U. guianensis* produces structurally dissimilar alkaloids compared to those found in *C. roseus*^5^. These results showed a precursor-directed biosynthesis strategy could provide the opportunity to rationally design both oxindole and vinca analogues, perhaps to improve their pharmacological properties. The biosynthetic pathways of POA and TOA from *U. guianensis* are in agreement with the previously described monoterpene indole alkaloid biosynthesis in *C. roseus*. After formation of the indole and terpenoid precursors (Fig. S46), secologanin and tryptamine precursors undergo a Mannich (Pictet-Spengler) reaction to form the tetrahydro-β-carboline strictosidine intermediate^24^. The intermediate dehydrogeissochizine^6^ is the key precursor for synthesis of both POA and TOA scaffolds. For POA, the ajmalicine (heteroyohimine-type alkaloid) likely undergoes an oxidation reaction which could be stereospecific, and yields intermediate I and II as described in *Strychnos*, *Aspidosperma* and *Iboga* alkaloids^25^. Our proposal involves a pinacol-pinacolone rearrangement, which is common in limonoids biosynthesis, as occurs in limonoids from *Khaya senegalensis*^26^. Intermediate II, which undergoes a rapid pinacol-pinacolone rearrangement, loses H_2_O from intermediate I, and is the key precursor of spirocyclic oxindole scaffold in order to produce either mitraphylline or isomitraphylline (POA). For TOA, the same mechanism observed for POA was proposed, mediated by dehydrogeissochizine which led to intermediate III, it then undergoes an oxidation step, yielding either rhynchophylline or isorhynchophylline from intermediate V.

Our results, together with previously reported data in *C. roseus*, seem to suggest that terpene indole and oxindole alkaloids are biosynthesized by similar pathways. Our studies showed that precursor-directed biosynthesis strategies using 5-methyl-, 7-methyl- and 6-fluoro-tryptamine precursors produce successful unnatural oxindole alkaloids in *U. guianensis* shoots. These results showed that a precursor-directed biosynthesis strategy could provide the opportunity to rationally design a broad variety of new molecules. Our work applies biosynthetic knowledge to advance the art of making new molecules which will inevitably trigger new potential drug candidates. We have also established the biosynthetic pathway of **1–4** which are derived from the MEP and shikimate pathways. This experiments also allowed us to establish the biosynthetic pathway for spirocyclic oxindole alkaloids with ^13^C-precursors for the first time in the literature.

## Methods

### Chemicals

The labeled precursors (1-^13^C-D-glucose, 1-^13^C-DL-glyceraldehyde, and methyl-^13^C-D-methionine) and analogue precursors (**a**-5-methyl-tryptamine, **b**-7-methyl-tryptamine and **c**-6-fluoro-tryptamine) were purchased from Sigma-Aldrich^®^. The 2-^13^C-tryptophan was obtained from Cambridge Isotope Laboratories, Inc.

### General procedure from *in vitro* shoot cultures of *U. guianensis*

The plant material was identified by Dr. Piero Giuseppe Delprete, of Herbier de Guyane, Institut de Recherche pour le Développement. A voucher specimen from *U. guianensis* was deposited in the Herbarium of Medicinal Plants at UNAERP (HPMU 2411), Ribeirão Preto, SP, Brazil. The collection of *U*. *guianensis* specimens investigated in this study was previously authorized by the Brazilian Council for the Administration and Management of Genetic Patrimony (CGEN) of the Brazilian Ministry of the Environment (MMA) via the National Council for Scientific and Technological Development (CGEN/MMA Process number: A2578BA). Disinfected explants (by 1% (w/v) cercobin and captan solutions) were added to MS medium^27^ with 3% (w/v) glucose, 0.2% phytogel^®^, 1 mM of benzylaminopurine (BAP), pH adjusted to 6.0 and maintained at 25 ± 2 °C (55–60% relative humidity with a 16-h photoperiod of 40 μmol m^−2^ s^−1^ intensity, provided by 85 W cool-white GE fluorescent lamps) and micropropagated every two months.

### Precusor-directed biosynthesis experiments

Typtamine analogues (**a–c**) were dissolved in 500 µL of DMSO (1 mM^5^), syringe filtered through a 0.2 µm filter to sterilize, and added to MS medium with 3% (w/v) glucose, 1 mM of BAP, pH adjusted to 6.0. *In vitro* plantlets (*n* = 50 shoots; 2 months old) were then transferred individually to the MS medium described above and maintained at 25 ± 2 °C for 30 days. After incubation, the aerial parts from fresh *U. guianensis* shoots were extracted with ethanol in order to obtain the crude extracts.

### HPLC-DAD and UPLC-DAD-QTOF analyses

The crude extracts were dissolved in ACN-H_2_O (8:2) and assessed by HPLC-DAD and UPLC-DAD-QTOF using modified chromatographic conditions from literature^28^. For UPLC-DAD-QTOF, samples were ionized by ESI with a MicrOTOF Bruker Daltonics mass spectrometer (Milford MA, USA). Mass spectra were obtained in positive ionization mode and the following parameters were applied: capillary energy of 3.5 kV, nitrogen was used as nebulization, drying gas (5.5 bar and 10 L/h), and temperature drying time of 220 °C. UPLC conditions were carried out using the isocratic system: 0–18′ (60:40; A:B), 18–32′ (50:50; A:B), and 32–40′ (100% of B %), 0.3 mL.min^−1^ flow rate and detection at 245 nm (A-triethylammonium acetate buffer 35 mM, 0.2% v/v, pH 6.9; B-ACN); and an Agilent Zorbax Eclipse XDB-C18 column: 3.5 µ, 4.6 × 150 mm. For HPLC-DAD, the samples were analyzed with the same chromatographic conditions described above, except for the flow rate, which was 0.8 mL.min^−1^.

### Isolation of unnatural oxindole alkaloids and NMR analysis

Crude extracts from aerial parts (0.8 g) were solubilized in ethanol-water solution (4:6), added with 500 mg of strong anionic resin (Dowex Marathon, Sigma-Aldrich) and submitted to magnetic agitation for 30 minutes. After this, a fraction enriched in oxindole alkaloid was obtained by a Dowex resin wash using ethanol-ammonium hydroxide (99.9:0.1) followed by magnetic agitation for 30 minutes and rota-evaporated at 37 °C. The fractions (8–10 mg) were then submitted to semi-preparative HPLC-DAD using the solvent system: 0–18′ (65:35; A:B), 18–32′ (50:50; A:B), and 32–40′ (100% of B %), 3 mL.min^−1^ flow rate, and detection at 245 nm (A-triethylammonium acetate buffer 35 mM, 0.2% v/v, pH 6.9; B-ACN); and an Agilent Zorbax Eclipse XDB-C18 column, 5 µ, 250 × 9.4 mm, to yield 7-methyl-isomitraphylline (**3b**, 0.7 mg) and 6-fluoro-isomitraphylline (**3c**, 0.8 mg). Isolated unnatural oxindole alkaloids were analyzed by NMR 1D and 2D (using DMSO-d_6_ as the solvent) with a 600 MHz Bruker Avance III HD equipped with a cryogenically cooled 5 mm dual probe optimized for ^13^C and ^1^H.

### ESI-QTOF-MS/MS analysis

The precursor ions of **3**, **3b** and **3c** were analyzed with a Triple TOF 5600 + DualSpray Ion Source AB SCiex (Massachusetts, U.S.A.) mass spectrometer. The ESI interface conditions were as follows: ion spray floating voltage = 4.0 kV, declustering potential = 70 V, gases 1 and 2 = 15 psi, and curtain gas = 25 psi. The sample was introduced into the mass spectrometer with the aid of a syringe pump coupled to the instrument operating at a flow rate of 5.0 μL/min. The tandem mass spectrometry experiments (MS/MS) with collision-induced dissociation were carried out by using N_2_ as collision gas on the selected precursor ion [M + H]^+^, at collision energy values ranging from 10 to 100 V. The mass analyzer was calibrated with a tuning solution that was supplied in the Chemical Standards Kit shipped with the system, to give a resolution of approximately 40,000. The mass data were processed with Analyst TF software.

### Feeding studies using ^13^C-precusors

The feeding experiments with 1-^13^C-D-glucose (3% m/v)^29^, 1-^13^C-DL-glyceraldehyde (1 mM), methyl-^13^C-D-methionine (0.3% m/v)^30^, and 2-^13^C-tryptophan (1 mM) were performed in liquid culture medium. *U. guianensis* plantlets (*n* = 30 shoots; 2 months old) were inoculated in liquid Murashige & Skoog medium with 1 mM of BAP supplemented with^13^-C-precursors (in four separate experiments), transferred into glass tubes (8.5 cm × 2.5 cm) containing 2.5 mL of liquid culture medium and incubated for 8 weeks. After this period, the aerial parts from *U. guianensis* shoots were extracted with ethanol for 12 h to obtain the crude extract.

### Isolation of ^13^C-labeled and non-labeled oxindole alkaloids (1–4) and NMR analysis

Chromatographic procedures for the isolation of ^13^C-labeled and non-labeled oxindole alkaloids were carried out using the procedure described above by anionic Dowex resin and semi-preparative HPLC to yield enriched mitraphylline (**1**, 2.0 mg derived from 1-^13^C-D-glucose, 2.0 mg derived from 2-^13^C-tryptophan, 2.0 mg derived from 1-^13^C-DL-glyceraldehyde, and 2.0 mg derived from methyl-^13^C-D-methionine); isomitraphylline (**3**, 3.0 mg derived from 1-^13^C-D-glucose, 3.0 mg derived from 2-^13^C-tryptophan, 3.0 mg derived from 1-^13^C-DL-glyceraldehyde, and 3.0 mg derived from methyl-^13^C-D-methionine); and isorhynchophylline (**4**, 2.0 mg derived from 1-^13^C-D-glucose). ^13^C NMR spectra of ^13^C-labeled, non-labeled, and unnatural oxindole alkaloids isolated were recorded with a 600 MHz Bruker Avance III HD using DMSO-d_6_. The relative ^13^C enrichments were obtained by comparing the relative intensity of both the labeled and non-labeled signals of oxindole alkaloids (**1**, **3** and **4**).

## Supplementary information

Supplementary Info
