# Supplementary material for: Unnatural spirocyclic oxindole alkaloids biosynthesis in Uncaria guianensis
Source: Sci Rep. 2019 Aug 5;9:11349. doi: 10.1038/s41598-019-47706-3 (PMC6683290; doi:10.1038/s41598-019-47706-3)
Supplement: Supplementary file 1 — Supplementary Info [file 41598_2019_47706_MOESM1_ESM.pdf]

Electronic Supporting Information for:

Unnatural spirocyclic oxindole alkaloids biosynthesis in *Uncaria guianensis*

Adriana A. Lopes[1*], Bianca Chioca[1†], Bruno Musquiari[1†], Eduardo J. Crevelin[2], Suzelei de C. França[1], Maria Fatima das G. Fernandes da Silva[3] and Ana Maria S. Pereira[1]

[1]Unidade de Biotecnologia, Universidade de Ribeirão Preto (UNAERP), Av. Costábile Romano, 2201, 14096-900, Ribeirão Preto, SP, Brazil.

[2]Faculdade de Filosofia, Ciências e Letras de Ribeirão Preto, Universidade de São Paulo (USP), Av. do Café s/n 14040-900, Ribeirão Preto, SP, Brazil.

[3]Centro de Ciências Exatas e de Tecnologia, Departamento de Química, Universidade Federal de São Carlos (UFSCar), Rod. Washington Luis s/n, 13565-905, São Carlos, SP, Brazil.

Correspondence and requests for materials should be addressed to A.A.L. (*e-mail: alopes@unaerp.br)

natural oxindole alkaloids

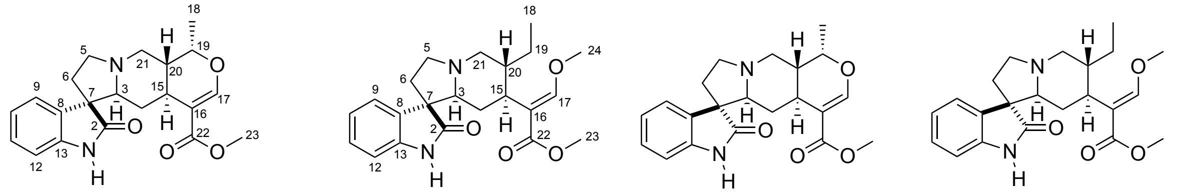

mitraphylline (1) **rhynchophylline (2)** **isomitraphylline (3)** **isorhynchophylline (4)**

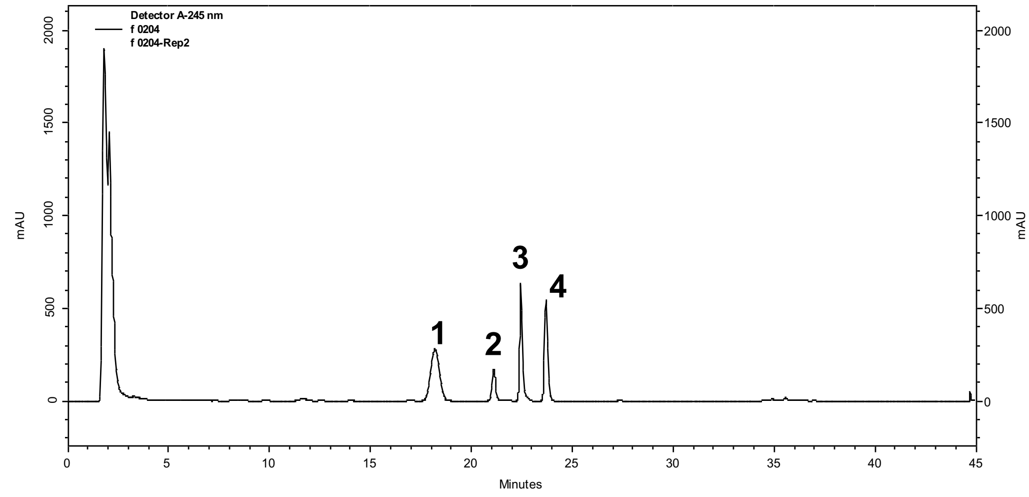

Figure S1. Chromatogram of methanolic extract from aerial parts of *U. guianensis* using HPLC conditions described in methods section.

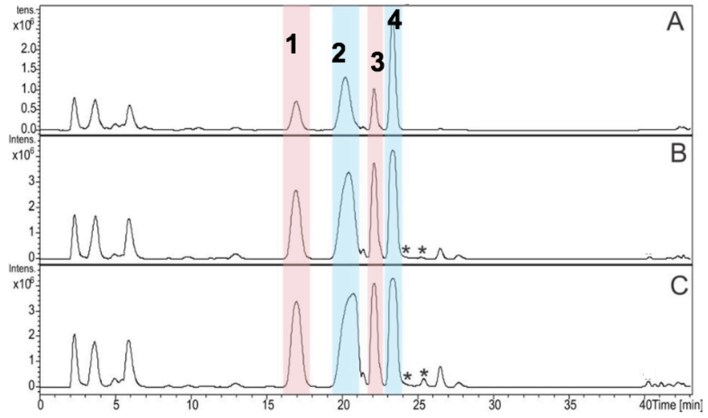

Figure S2. Chromatogram of control extract (A) and extracts obtained after supplementation with **a**-5-methyl-tryptamine (B) and **b**-7-methyl-tryptamine (C), an asterisk is used to represent unnatural oxindole alkaloids biosynthesis.

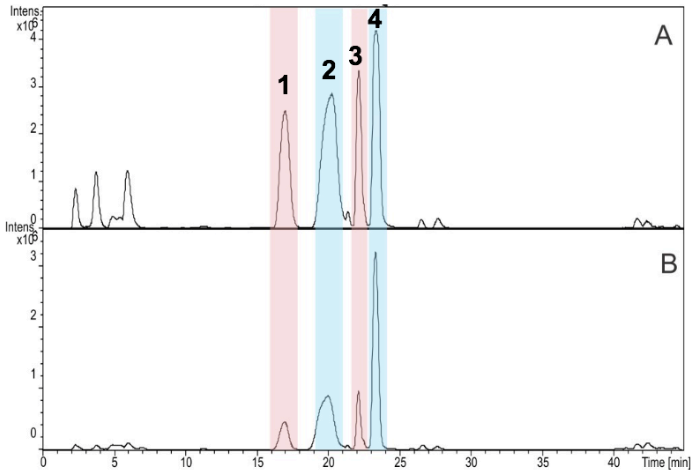

Figure S3. Chromatogram of control extract (A) and extracts obtained after supplementation with **c**-6-fluoro-tryptamine (B), the unnatural oxindole alkaloids co-eluted with natural oxindole alkaloids (**1-4**).

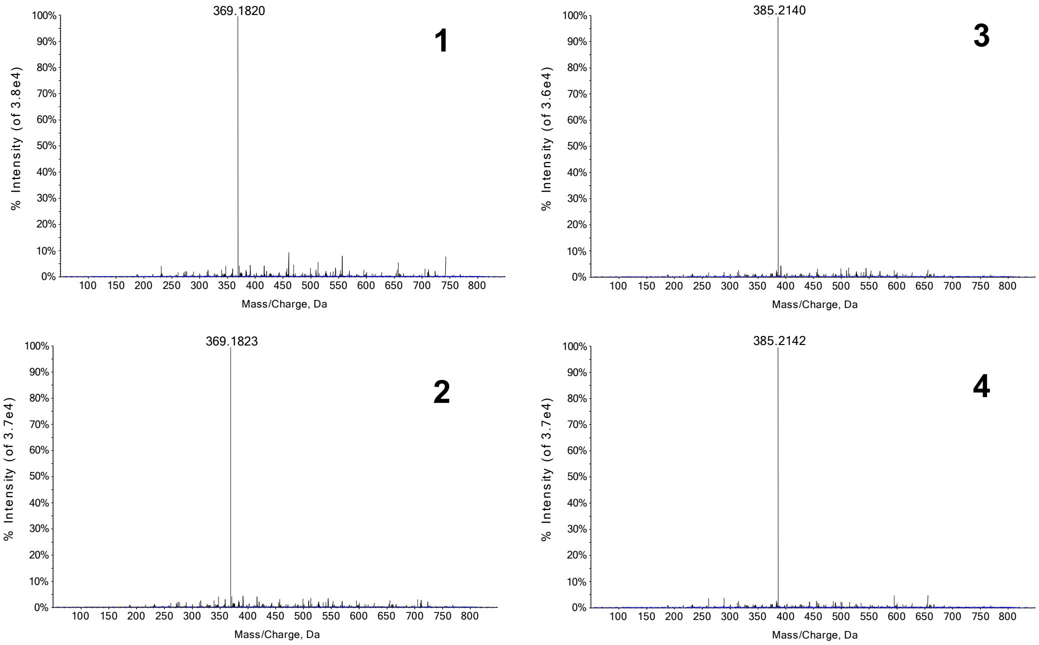

Figure S4. ESI-QTOF-MS/MS of natural oxindole alkaloids (**1-4**).

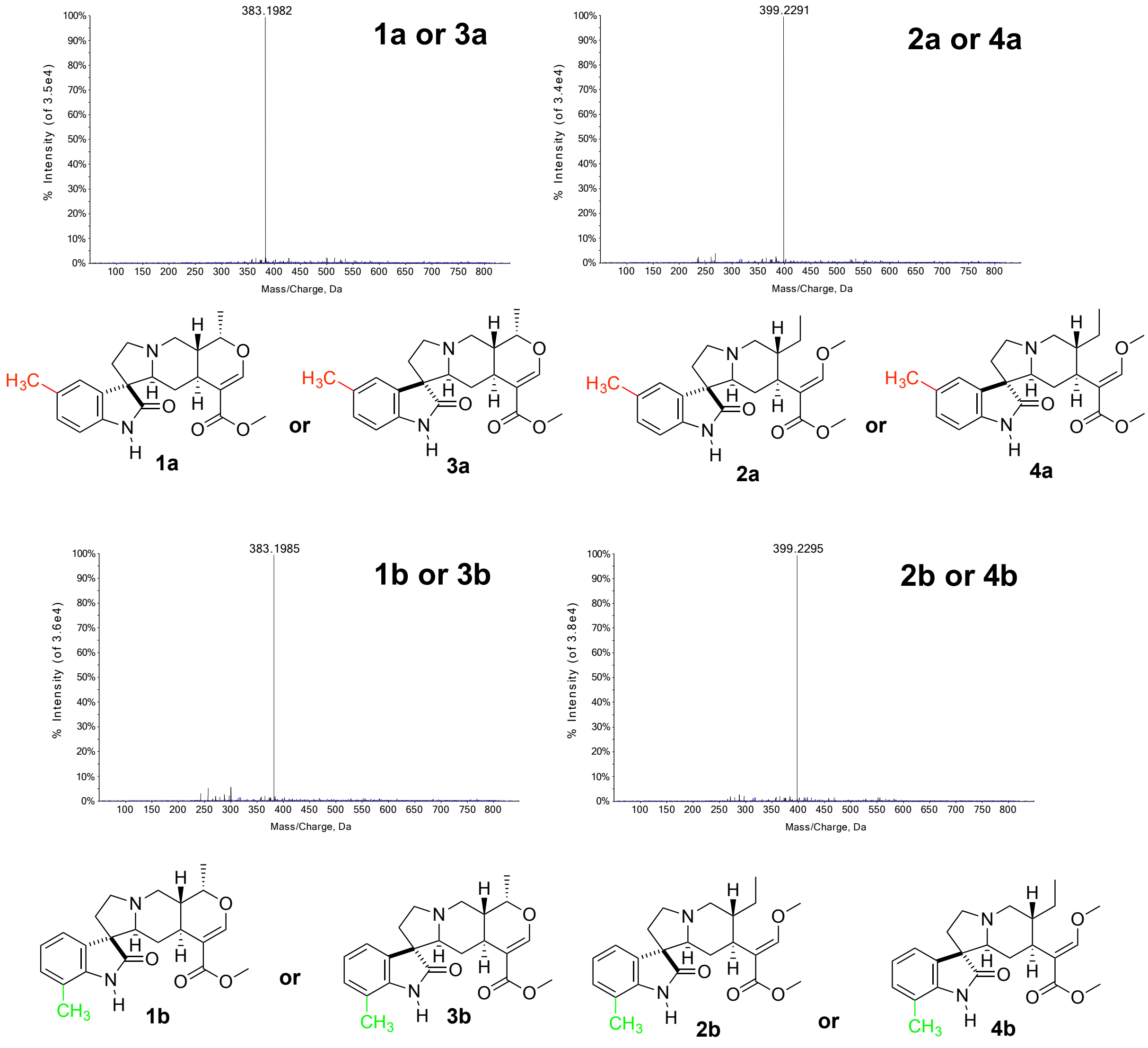

Figure S5. ESI-QTOF-MS/MS of methylated oxindole alkaloids produced by *U. guianensis* plantlets.

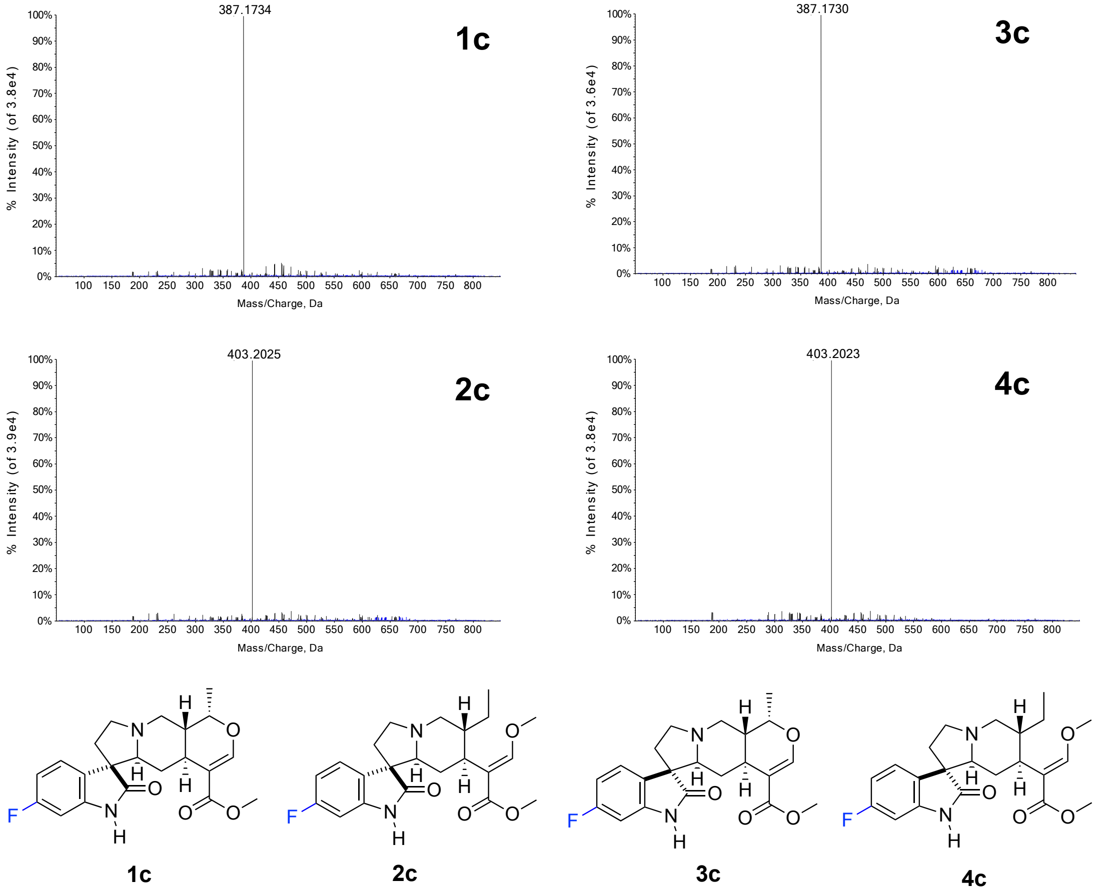

Figure S6. ESI-QTOF-MS/MS of fluorated oxindole alkaloids produced by *U. guianensis* plantlets.

Table S1. ESI-QTof-MS data of compounds.

**Compounds**	***Observed m/z***	***Calculated m/z***	**error (ppm)**	**Molecular Formula**
mitraphylline (**1**)	369.1820	369.1814	1.6	$[C_{21}H_{24}N_2O_4+H]^+$
isomitraphylline (**3**)	369.1823	369.1814	2.4	$[C_{21}H_{24}N_2O_4+H]^+$
rinchophylline (**2**)	385.2140	385.2127	3.4	$[C_{22}H_{23}N_2O_4+H]^+$
isorinchophylline (**4**)	385.2142	385.2127	3.9	$[C_{22}H_{23}N_2O_4+H]^+$
5-methyl-mitraphylline/5-methyl-isomitraphylline (**1a/3a**)	383.1982	383.1970	3.1	$[C_{22}H_{26}N_2O_4+H]^+$
5-methyl-rinchophylline/5-methyl-isorinchophylline (**2a/3a**)	399.2291	399.2284	1.8	$[C_{23}H_{30}N_2O_4+H]^+$
7-methyl-mitraphylline/7-methyl-isomitraphylline (**1b/3b**)	383.1985	383.1970	3.9	$[C_{22}H_{26}N_2O_4+H]^+$
7-methyl-rinchophylline/7-methyl-isorinchophylline (**2b/3b**)	399.2295	399.2284	2.8	$[C_{23}H_{30}N_2O_4+H]^+$
6-fluor-mitraphylline (**1c**)	387.1734	387.1720	3.6	$[C_{21}H_{23}FN_2O_4+H]^+$
6-fluor-isomitraphylline (**3c**)	387.1730	387.1720	2.6	$[C_{21}H_{23}FN_2O_4+H]^+$
6-fluor-rinchophylline (**2c**)	403.2025	403.2033	-2.0	$[C_{22}H_{27}FN_2O_4+H]^+$
6-fluor-isorinchophylline (**4c**)	403.2023	403.2033	-2.5	$[C_{22}H_{27}FN_2O_4+H]^+$

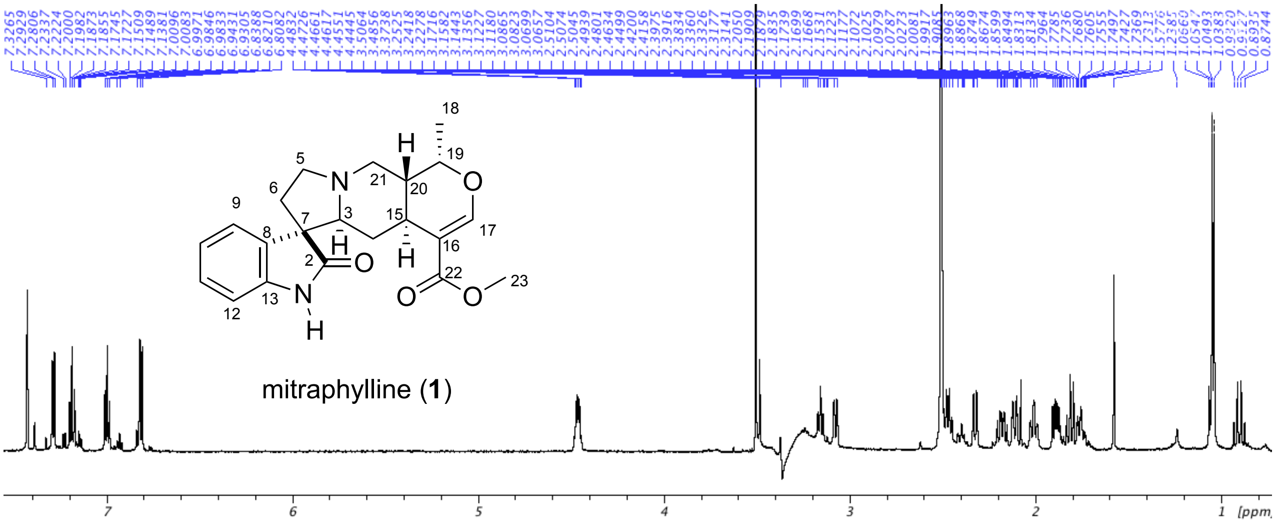

Figure S7. ^{1}H-NMR spectrum of **1** (600 MHz, DMSO-d_6).

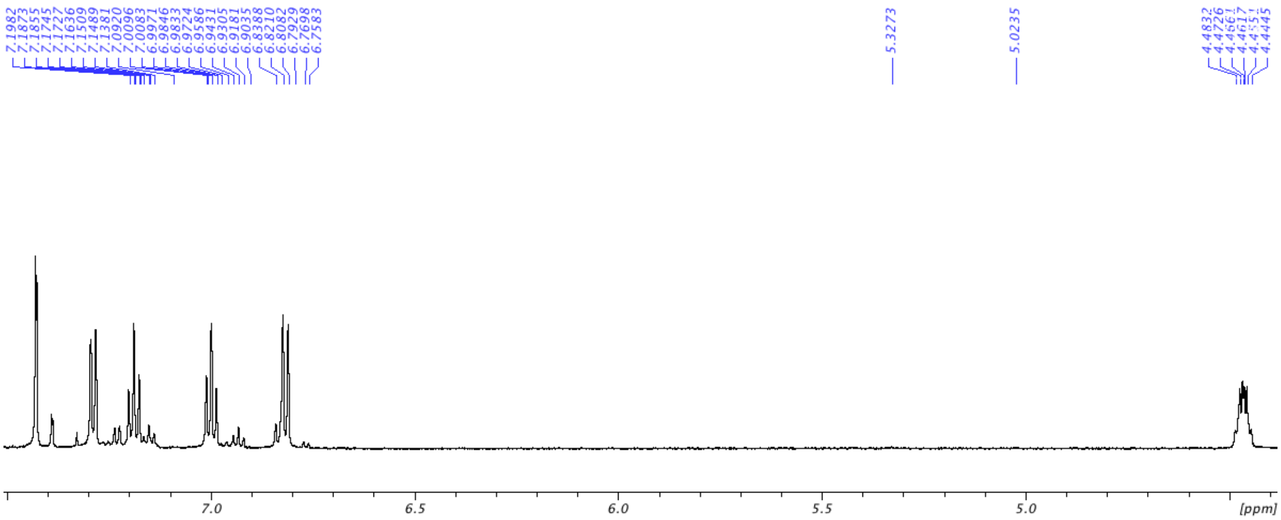

Figure S8. ^{1}H-NMR spectrum (expanded view) of **1** (600 MHz, DMSO-d_6).

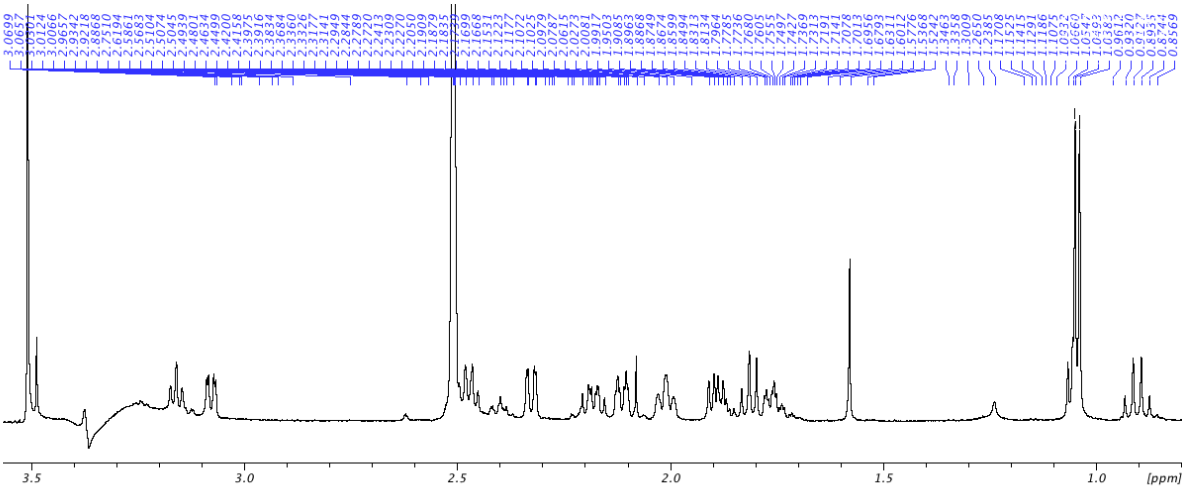

Figure S9. ^{1}H-NMR spectrum (expanded view) of **1** (600 MHz, DMSO-d_6).

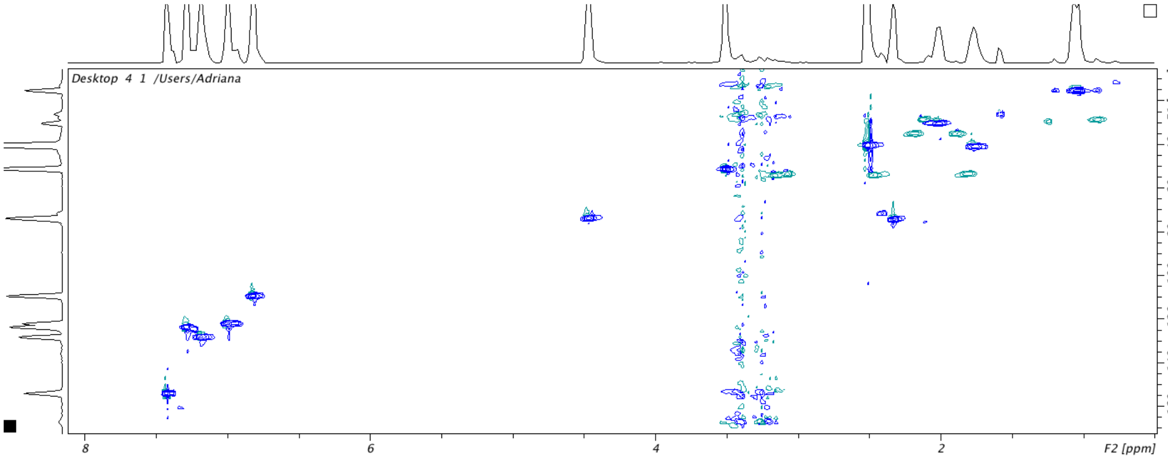

Figure S10. HSQC spectrum of **1** in DMSO-d$_6$ (600 MHz).

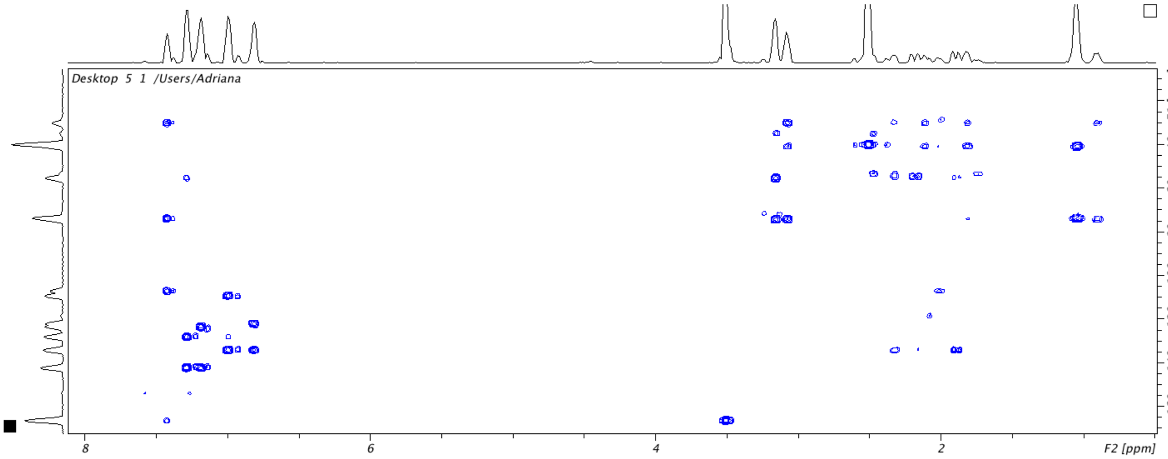

Figure S11. HMBC spectrum of **1** in DMSO-d$_6$ (600 MHz).

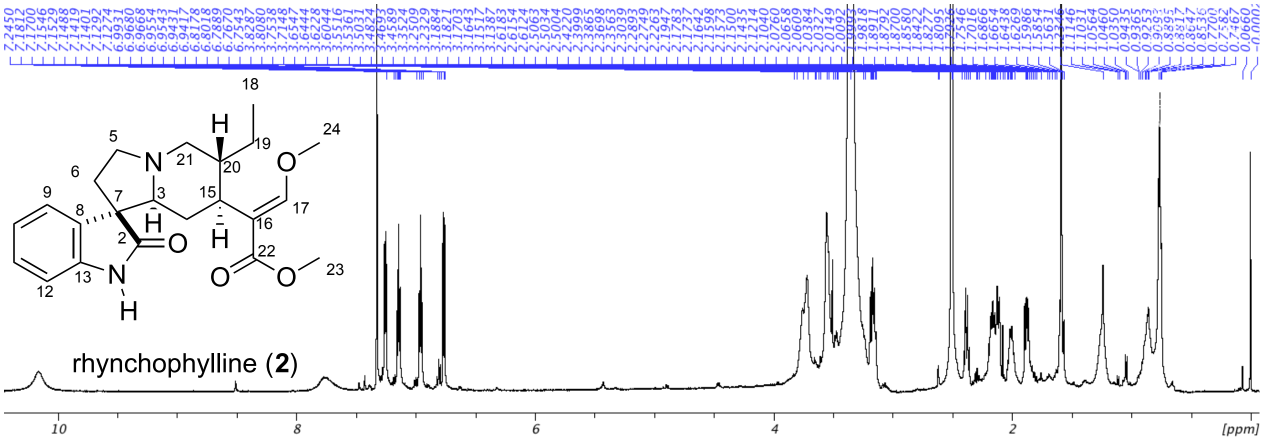

Figure S12. ^{1}H-NMR spectrum of **2** (600 MHz, DMSO-d$_6$).

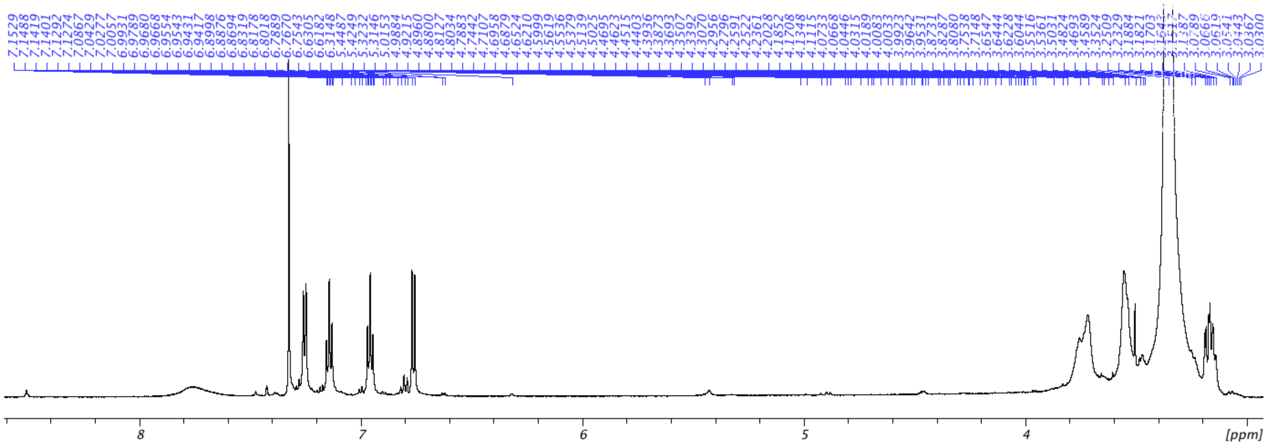

Figure S13. ^{1}H-NMR spectrum (expanded view) of **2** (600 MHz, DMSO-d_6).

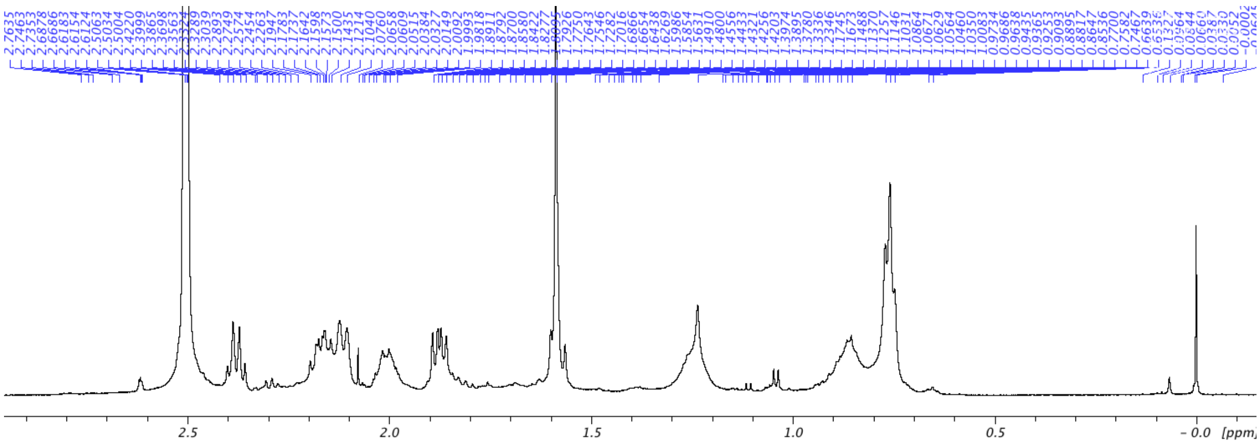

Figure S14. ^{1}H-NMR spectrum (expanded view) of **2** (600 MHz, DMSO-d_6).

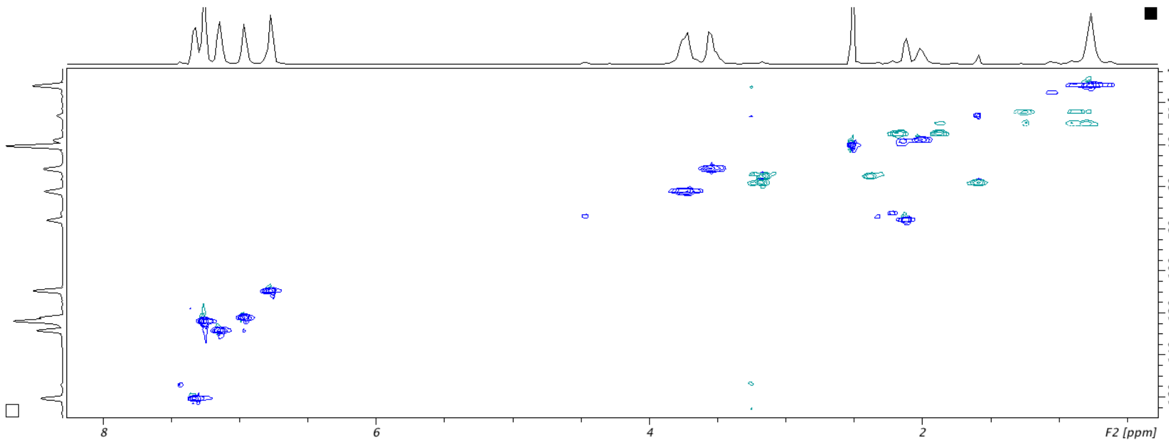

Figure S15. HSQC spectrum of **2** in DMSO-d_6 (600 MHz).

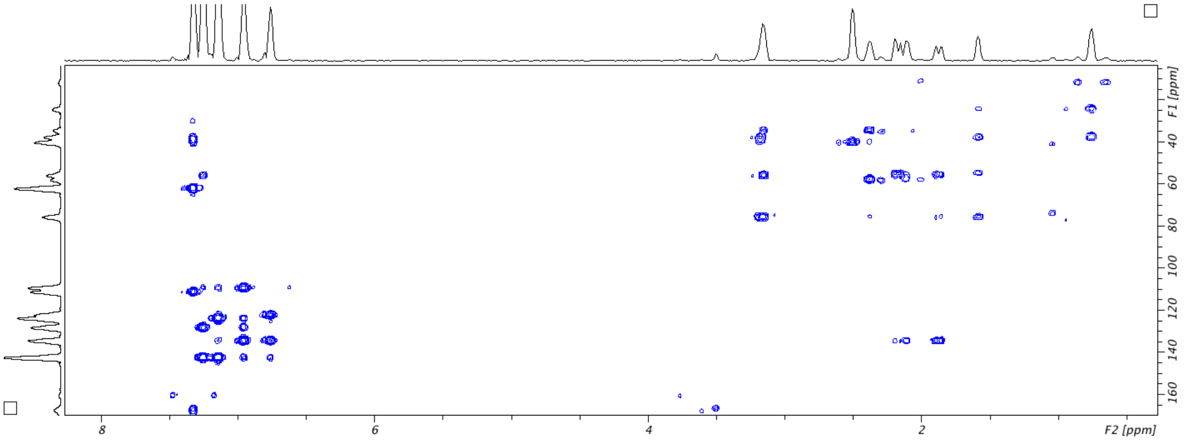

Figure S16. HMBC spectrum of **2** in DMSO-d$_6$ (600 MHz).

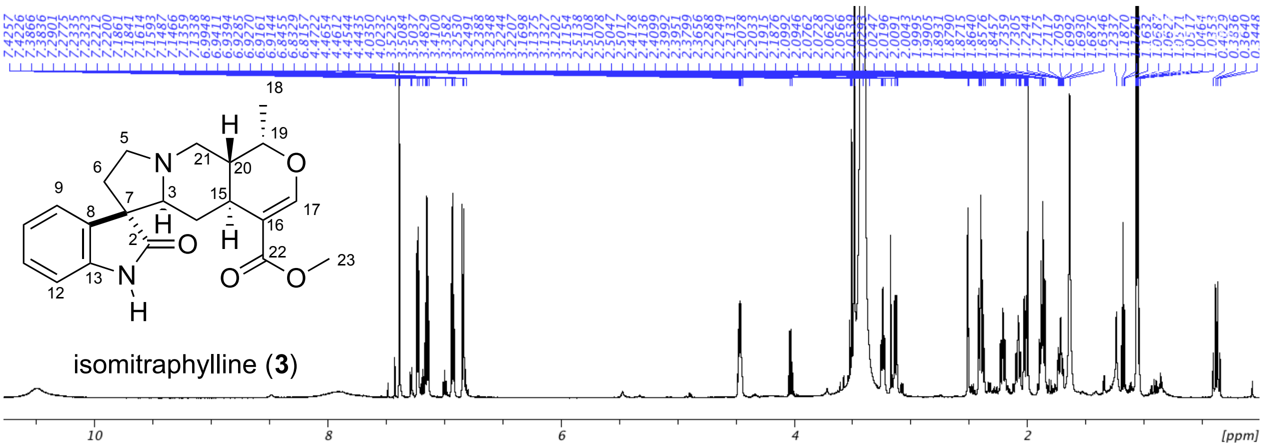

Figure S17. ^{1}H-NMR spectrum of **3** (600 MHz, DMSO-d$_6$).

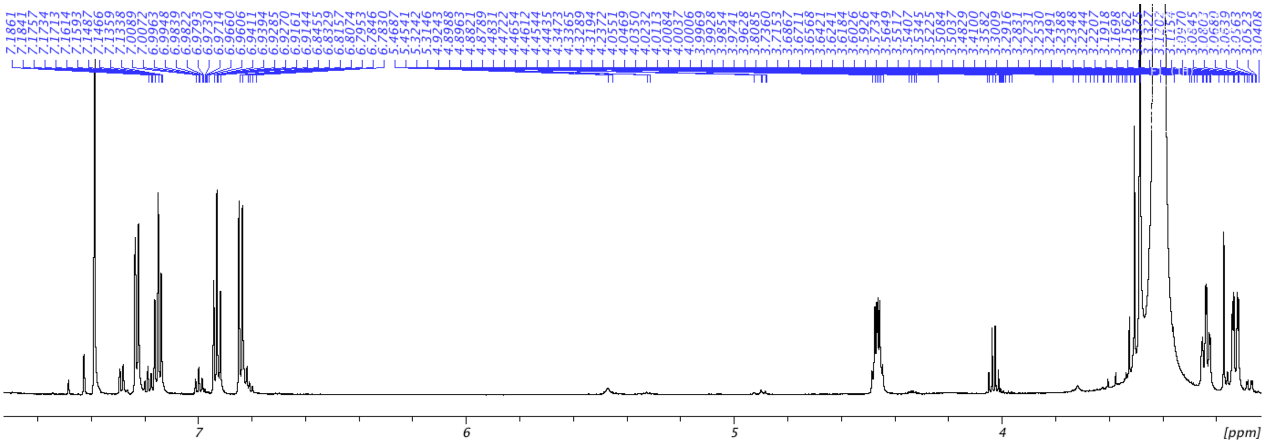

Figure S18. ^{1}H-NMR spectrum (expanded view) of **3** (600 MHz, DMSO-d$_6$).

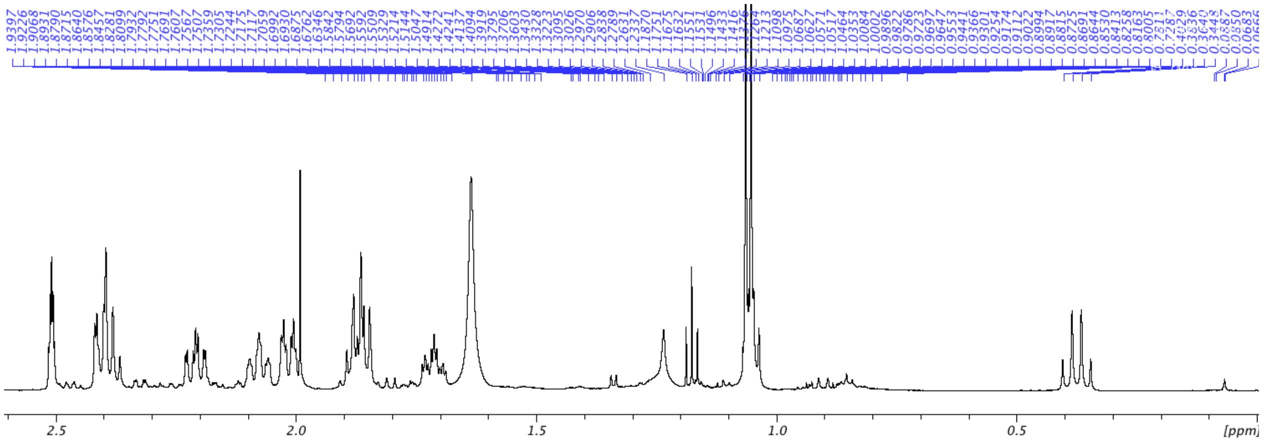

Figure S19. ^{1}H-NMR spectrum (expanded view) of **3** (600 MHz, DMSO-d_6).

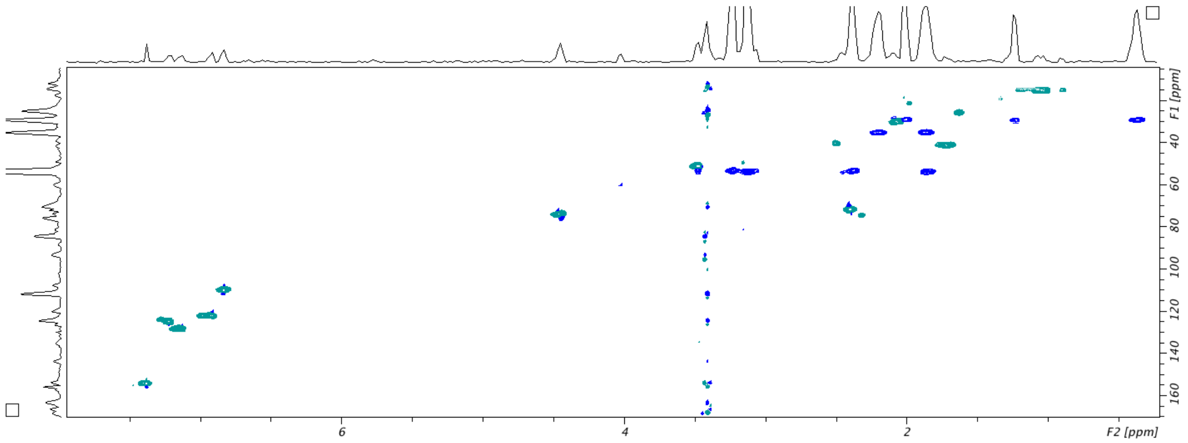

Figure S20. HSQC spectrum of **3** in DMSO-d_6 (600 MHz).

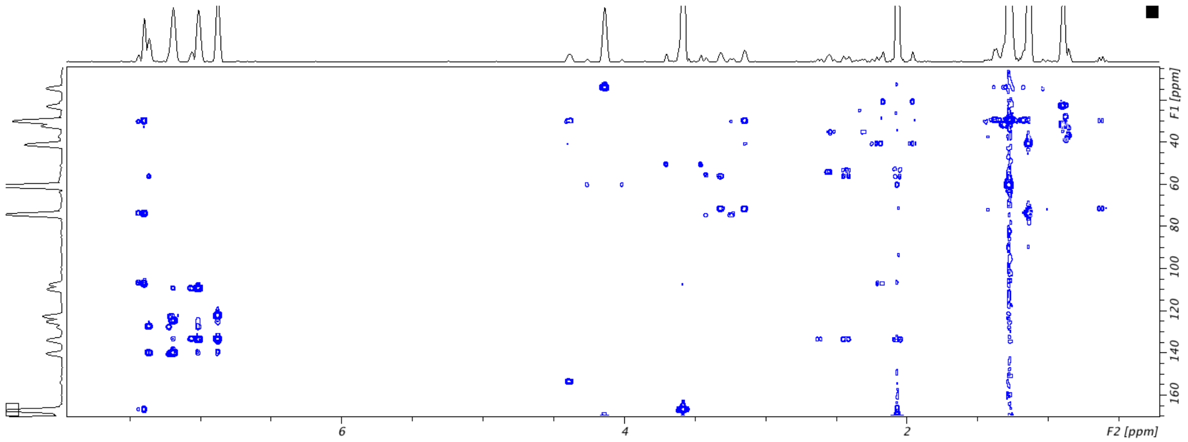

Figure S21. HMBC spectrum of **3** in DMSO-d_6 (600 MHz).

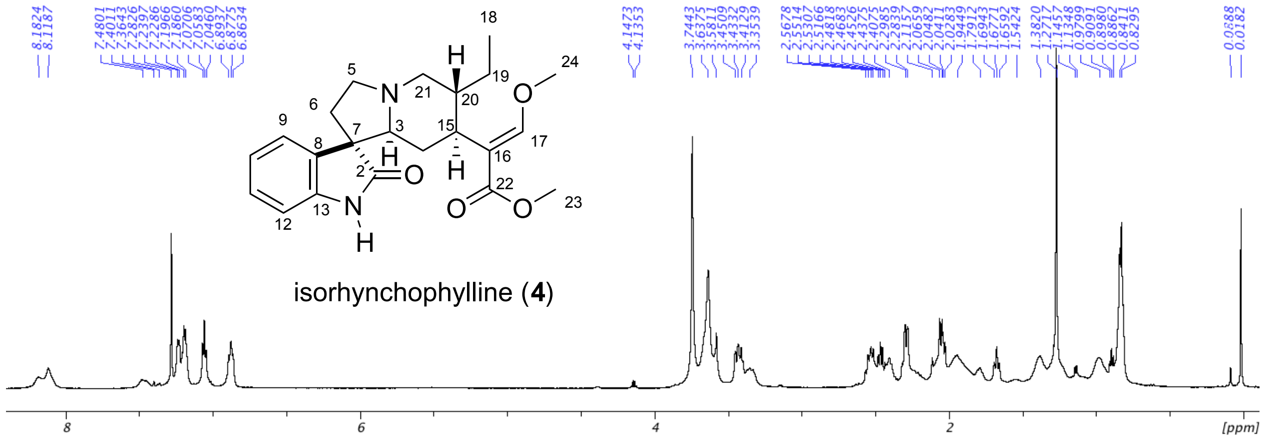

Figure S22. ^{1}H-NMR spectrum of **4** (600 MHz, DMSO-d_6).

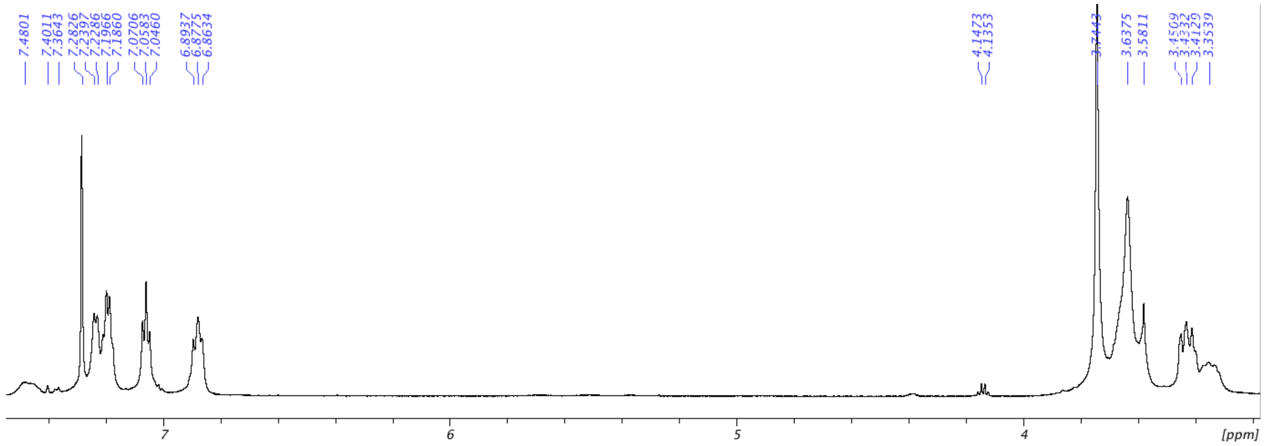

Figure S23. ^{1}H-NMR spectrum (expanded view) of **4** (600 MHz, DMSO-d_6).

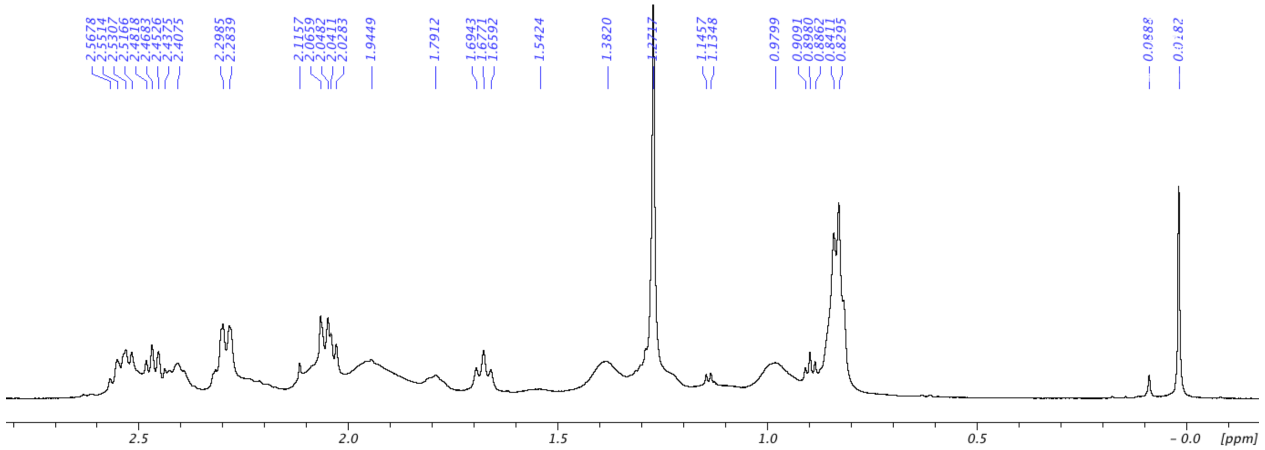

Figure S24. ^{1}H-NMR spectrum (expanded view) of **4** (600 MHz, DMSO-d_6).

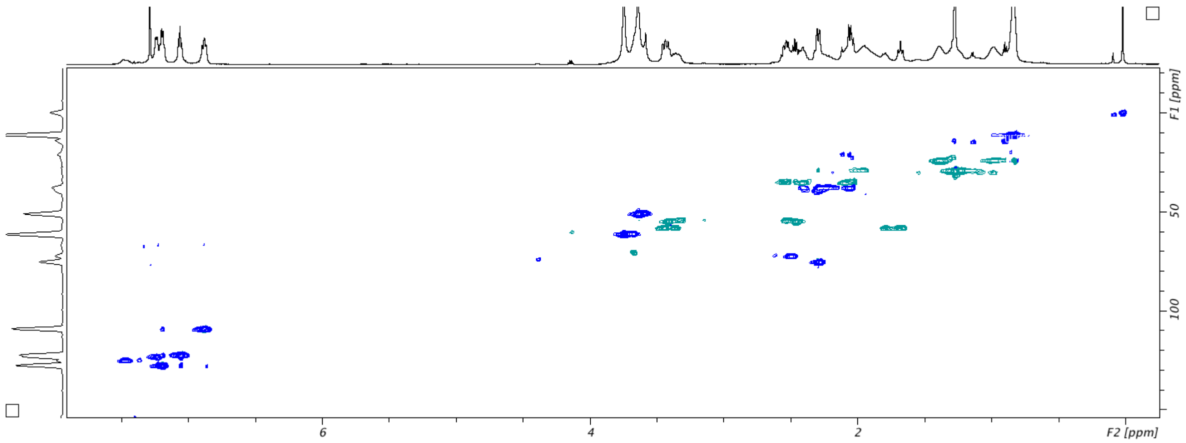

Figure S25. HSQC spectrum of **4** in DMSO-d$_6$ (600 MHz).

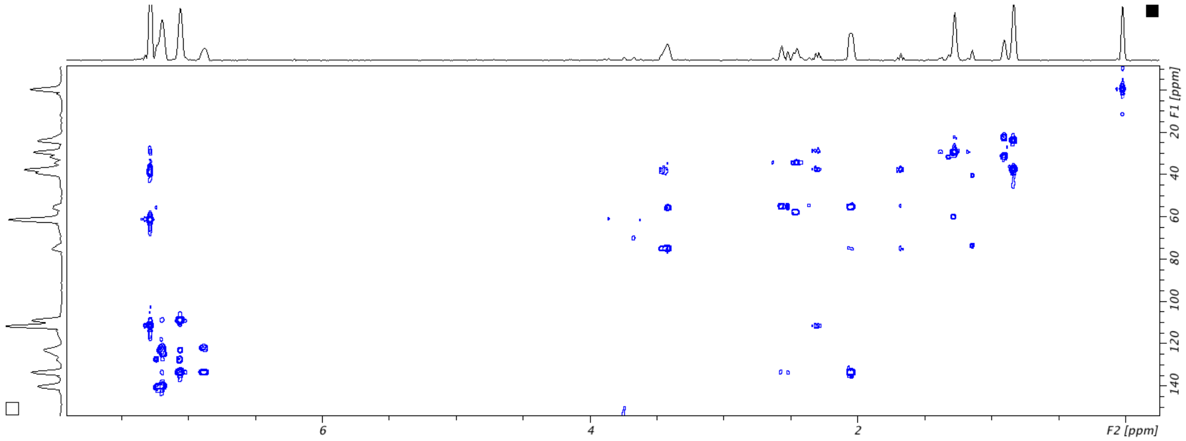

Figure S26. HMBC spectrum of **4** in DMSO-d$_6$ (600 MHz).

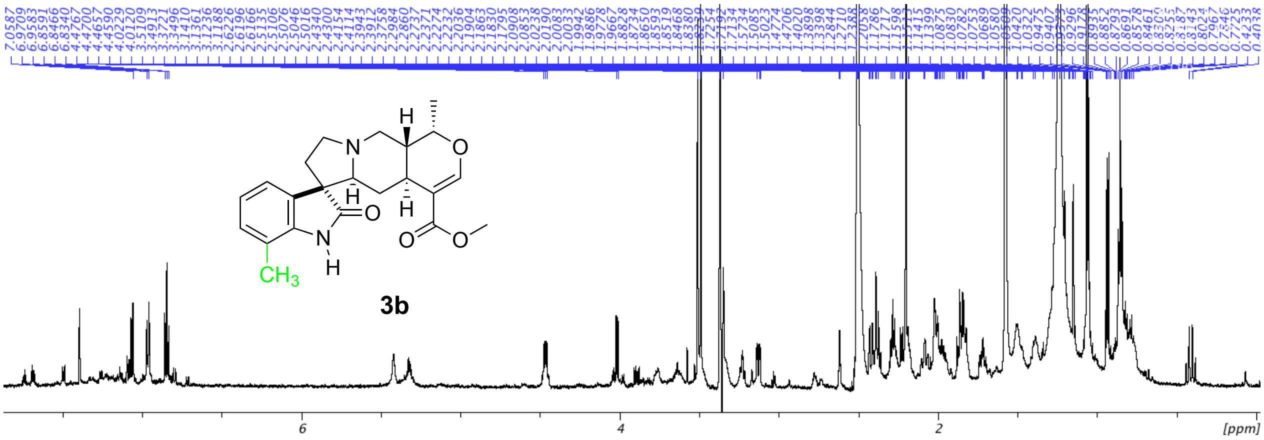

Figure S27. ^{1}H-NMR spectrum of **3b** (600 MHz, DMSO-d$_6$).

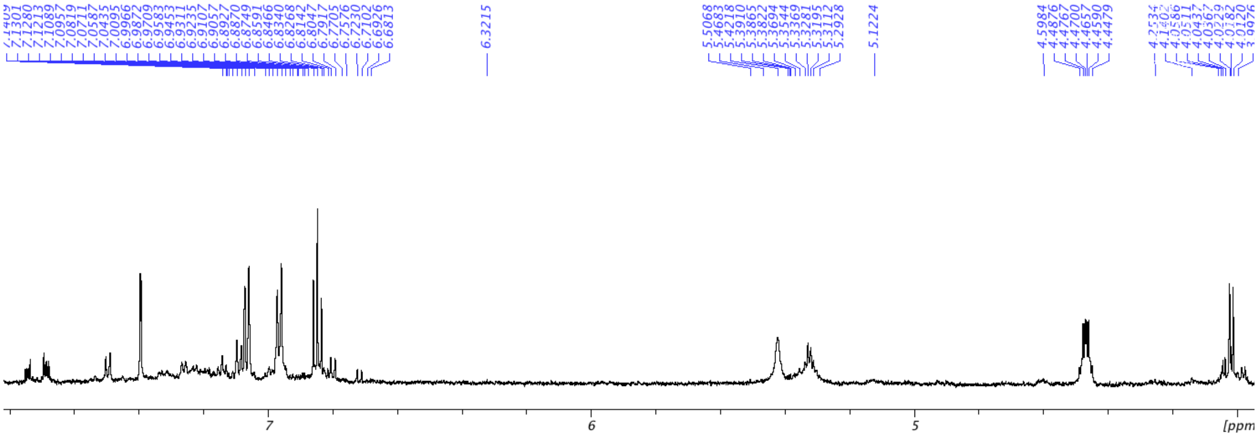

Figure S28. ^{1}H-NMR spectrum (expanded view) of **3b** (600 MHz, DMSO-d$_6$).

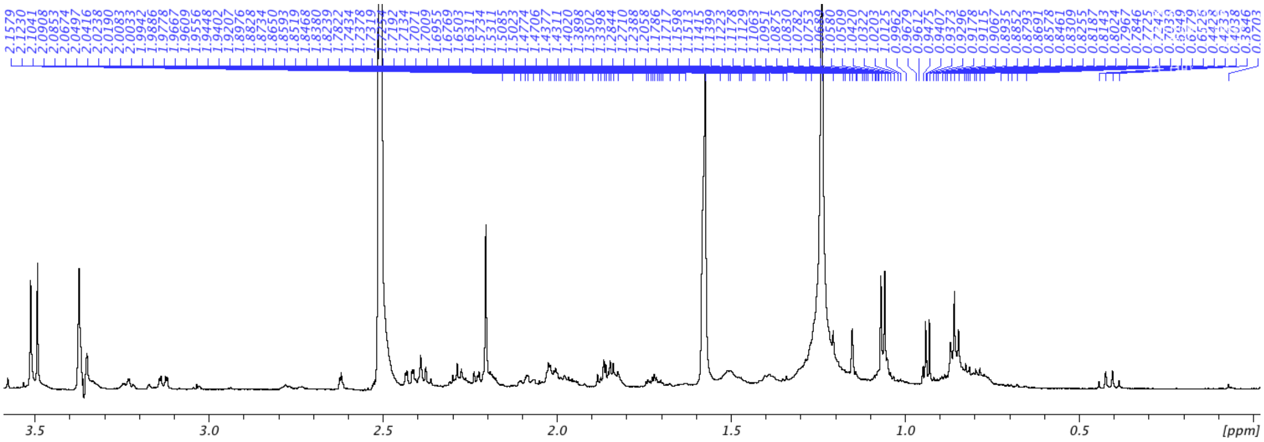

Figure S29. ^{1}H-NMR spectrum (expanded view) of **3b** (600 MHz, DMSO-d$_6$).

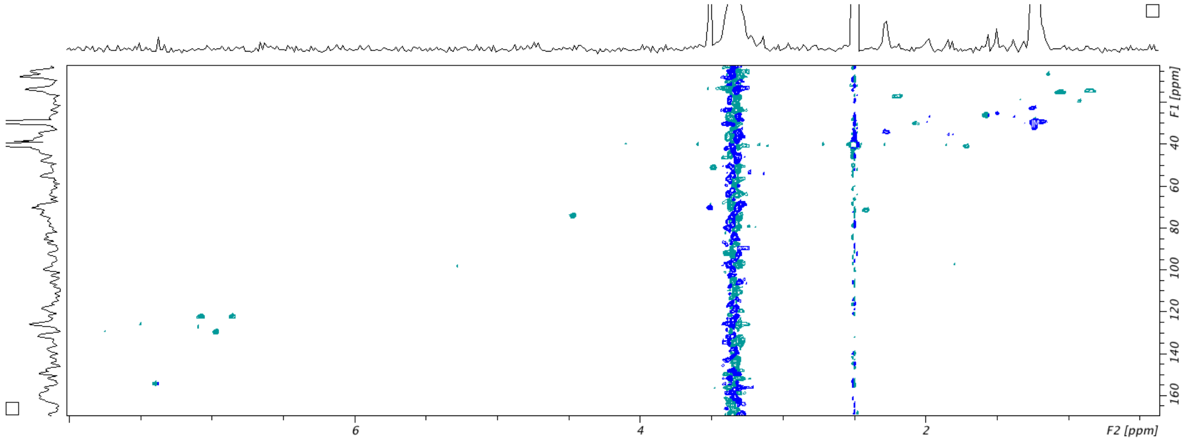

Figure S30. HSQC spectrum of **3b** in DMSO-d$_6$ (600 MHz).

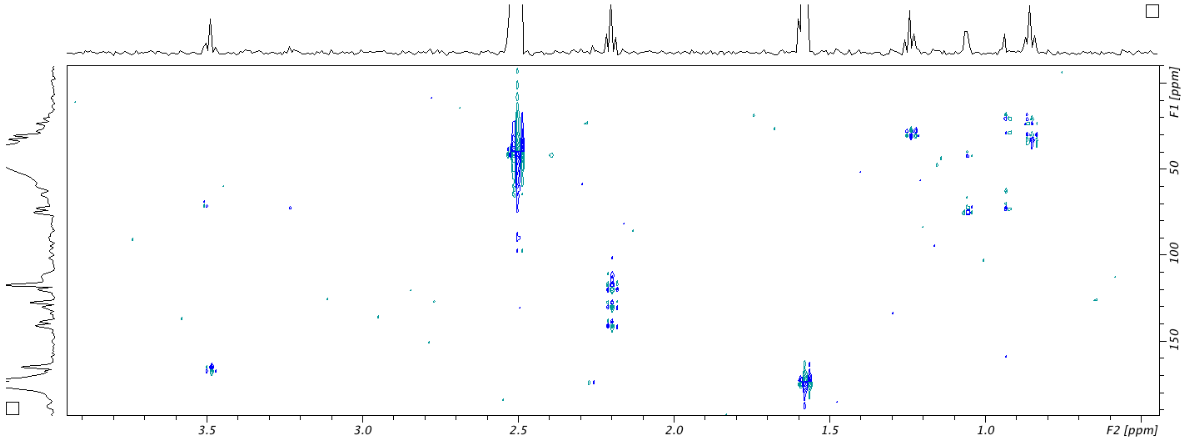

Figure S31. HMBC spectrum of **3b** in DMSO-d$_6$ (600 MHz).

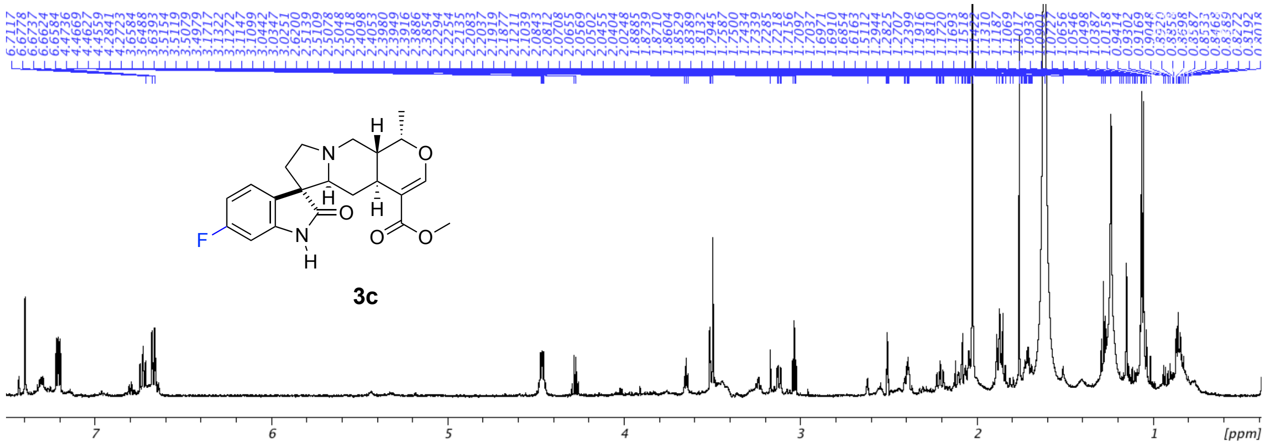

Figure S32. ^{1}H-NMR spectrum of **3c** (600 MHz, DMSO-d$_6$).

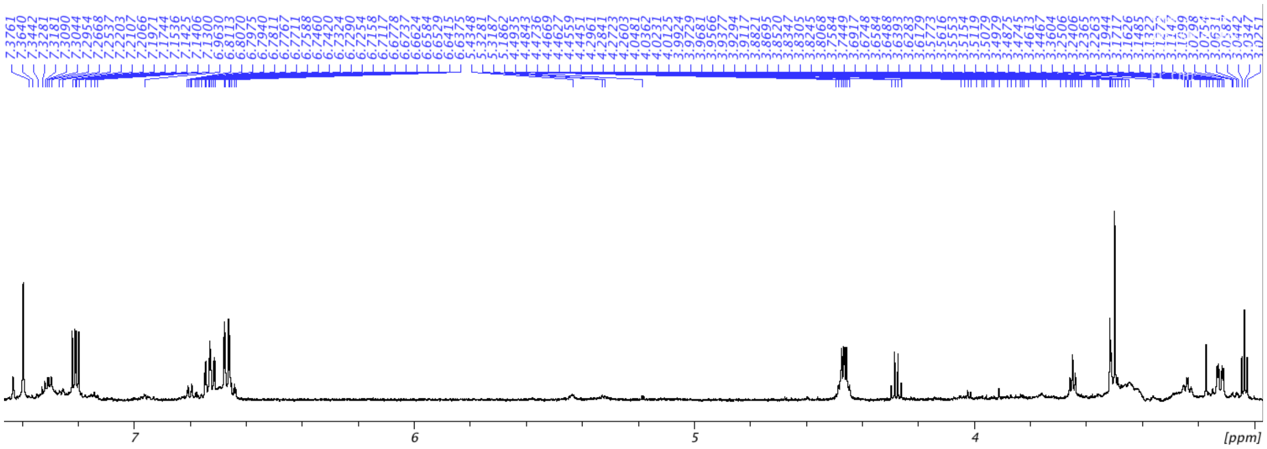

Figure S33. ^{1}H-NMR spectrum (expanded view) of **3c** (600 MHz, DMSO-d_6).

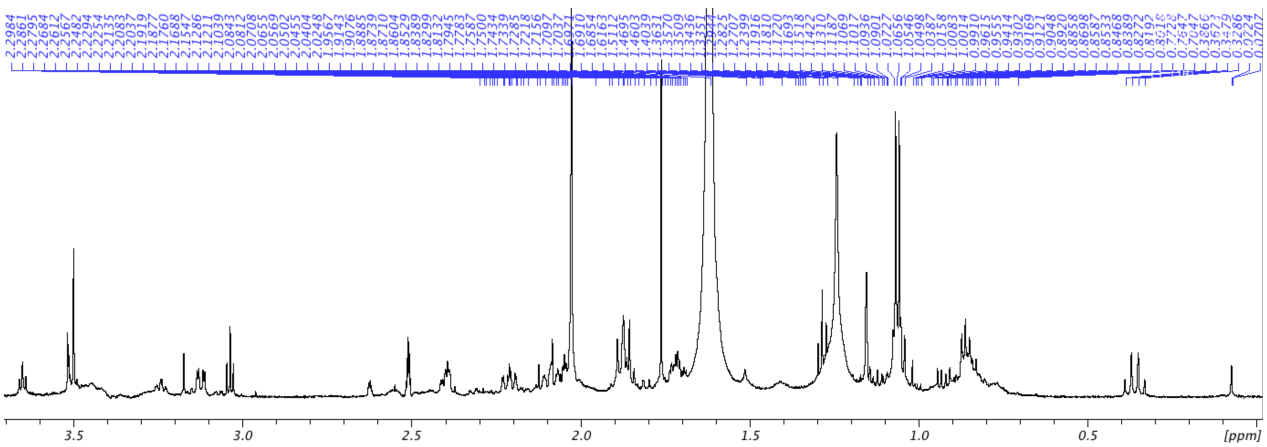

Figure S34. ^{1}H-NMR spectrum (expanded view) of **3c** (600 MHz, DMSO-d_6).

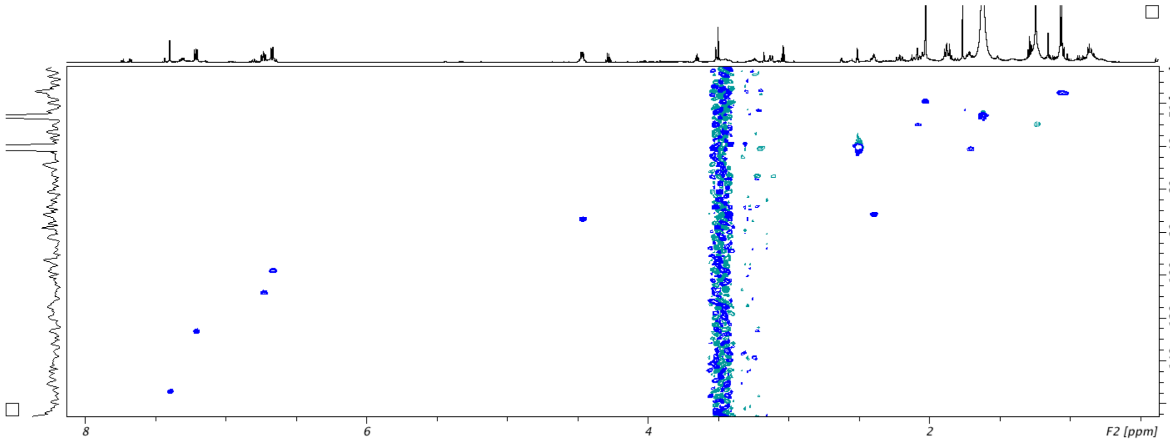

Figure S35. HSQC spectrum of **3c** in DMSO-d_6 (600 MHz).

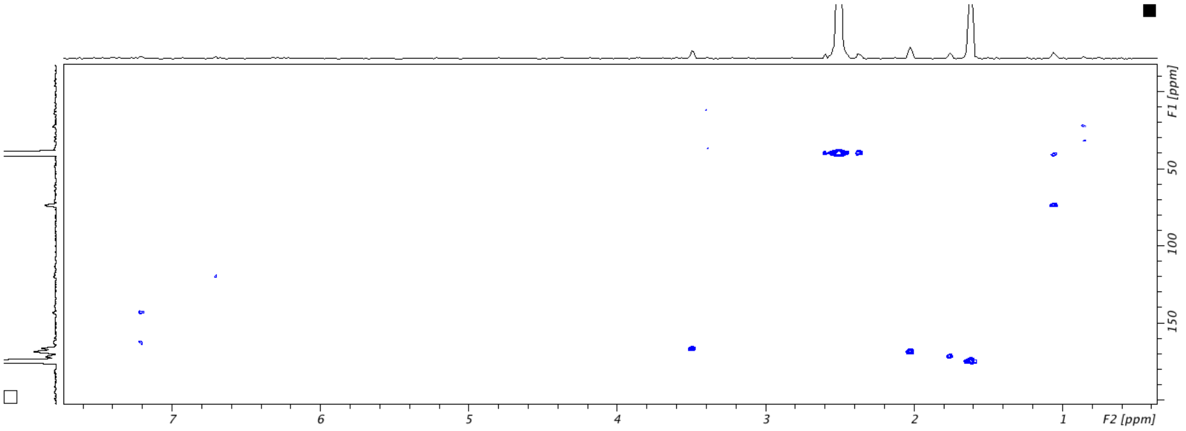

Figure S36. HMBC spectrum of **3c** in DMSO-d$_6$ (600 MHz).

Table S2. ^{1}H and ^{13}C NMR data for isomitraphylline (**3**), 7-methyl-isomitraphylline (**3b**) and 6-fluoro-isomitraphylline (**3c**) (600 MHz; DMSO-d_6)

	**3**[1]		**3b**		**3c**	
	**RMN de ^{1}H**	**RMN de ^{13}C**	**RMN de ^{1}H**	***g*HMQC***	**RMN de ^{1}H**	***g*HMQC***
**2**	-	181.6	-	*no*	-	*no*
**3**	2.50 m	71.6	2.41 m	71.8	2.39 m (1H)	71.8
**5**	2.50 m; 3.19 t (J=6,8 Hz)	53.3	3.2 m (2H)	53.5	*no*	*no*
**6**	2.0 m; 2.3 m	35.3	2.28 m; 1.57 m (2H)	34.1	1.85 m	35.3
**7**	-	56.4	-	*no*	-	58.0
**8**	-	133.7	-	*no*	-	129.3
**9**	7.27 d (J=7.5 Hz)	124.7	7.06 d (J= 7.5 Hz; 1H)	122.1	7.21 dd (J= 8.4; **6.0** Hz; 1H)	126.4
**10**	6.90 ddd (J= 7.5; 7.6; 0.9 Hz)	122.2	6.8 t (J= 7.5 Hz; 1H)	122.1	6.72 ddd (J= **10.0**; 7.8; 2.4 Hz; 1H)	107.8
**11**	7.09 ddd (J= 7.6; 7.7; 1.3 Hz)	127.4	6.96 d (J= 7.5 Hz; 1H)	129.3	-	162.6
**12**	6.81 d (J= 7.7 Hz)	109.6	-	119.0	6.68 dd (J= **9.0**; 2.4 Hz; 1H)	97.9
**13**	-	140.3	-	140.0	-	142.0
**14**	0.54 q (J=11.6 Hz), 2.5 m	29.1	0.93 m	19.4	0.36 q (J=11.6 Hz; 1H), 2.03 m	29.2
**15**	1.80 m; 2.10 m	29.9	2.07 m (1H)	30.7	2.07 m (1H)	30.0
**16**	-	107.2	-	*no*	-	108.0
**17**	7.31 d (J=1.4 Hz)	153.7	7.39 d (J= 1.5 Hz, 1H)	154.2	7.39 d (J= 1.8 Hz, 1H)	154.1
**18**	1.05 d (J=6.6 Hz)	14.8	1.05 d (J= 6.6 Hz, 3H)	15.1	1.05 d (J= 6.6 Hz, 3H)	15.0
**19**	4.30 m	73.8	4.47 m (1H)	74.2	4.46 m (1H)	73.8
**20**	1.85 m	40.8	1.71 m (1H)	41.08	1.71 m	41.2
**21**	1.8 t (J=10.4 Hz); 3.10 dd (J= 10.4; 2.0 Hz)	54.2	3.13 m; 1.8 m (2H)	54.4	3.12 dd (*J*= 10.0; 2.0 Hz, 1H); 1.85 m	53.7
**22**	-	167.0	-	*no*	-	166.6
**23**	3.49 s	50.7	3.48 s	51.0	3.49 s	*no*
**24**	-	-	2.20 s (3H)	17.2	-	-

no- "non-observed"
*^{13}C data based in *g*HMQC
The coupling constant in bold is related to **H-F**.

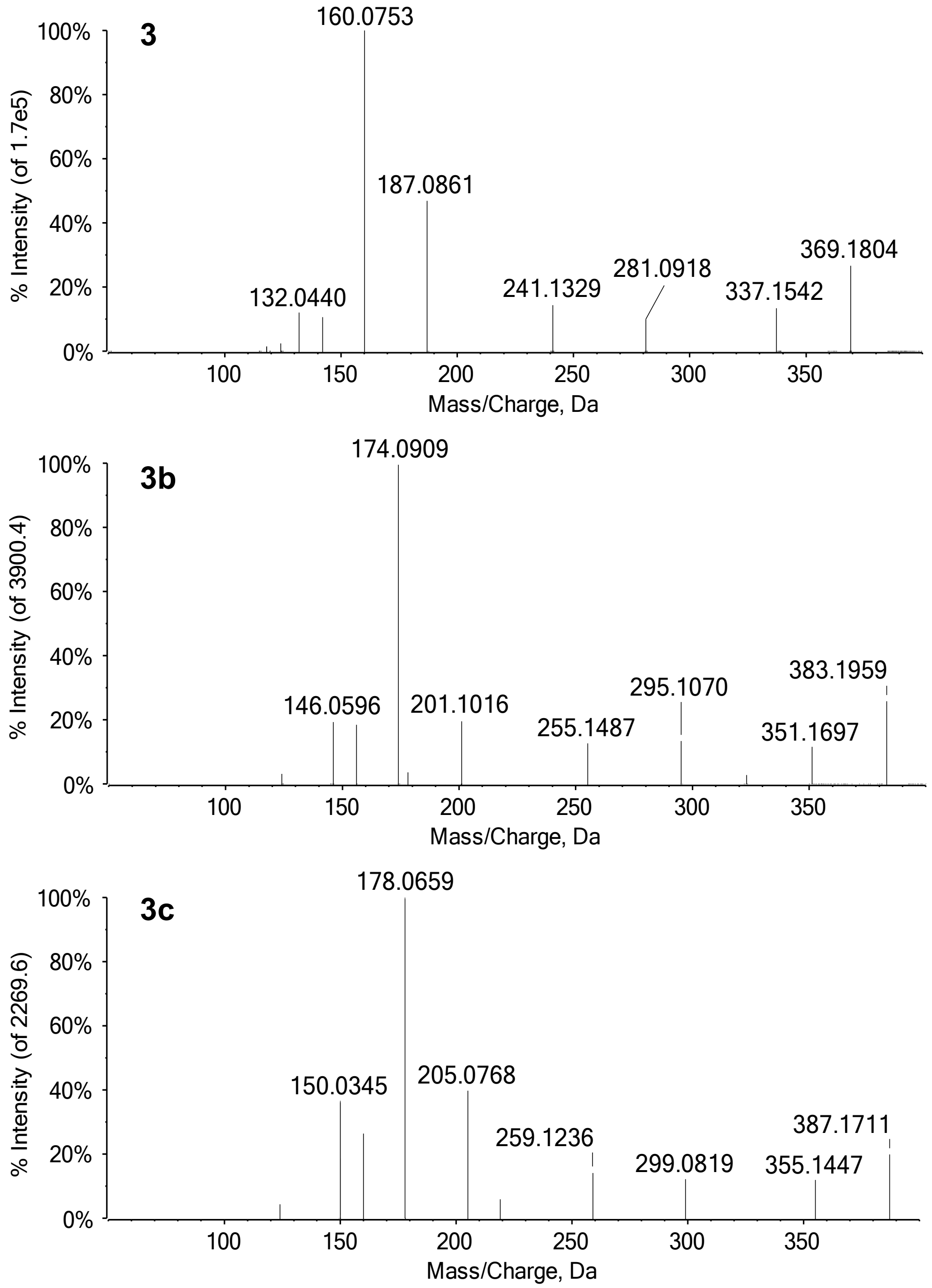

Figure S37. ESI-MS/MS spectra of **3**, **3b** and **3c** obtained on an ESI-QTOF.

Table S3. ESI-QTOF-MS/MS data of oxindole alkaloids **3, 3b** and **3c**.

Observed *m/z*[a,b]	Calculated *m/z*	Error (ppm)	Formula of the ion	Assignment
**3**				
369.1804 (25)	369.1809	+1.4	$C_{21}H_{25}N_2O_4^+$	**A**
337.1542 (13)	337.1547	+1.5	$C_{20}H_{21}N_2O_3^+$	**B** (**A**–CH_3OH)
281.0918 (10)	281.0921	+1.1	$C_{16}H_{13}N_2O_3^+$	**C** (**B**–C_4H_8)
241.1329 (15)	241.1335	+2.5	$C_{15}H_{17}N_2O^+$	**D** (**A**–$C_6H_8O_3$)
187.0861 (48)	187.0866	+2.7	$C_{11}H_{11}N_2O^+$	**E** (**D**–C_4H_6)
160.0753 (100)	160.0757	+2.5	$C_{10}H_{10}NO^+$	**F** (**E**–HCN)
142.0646 (11)	142.0651	+3.5	$C_{10}H_8N^+$	**G** (**F**–H_2O)
132.0440 (13)	132.0444	+3.0	$C_8H_6NO^+$	**H** (**E**–C_2H_4–HCN)
**3b**				
383.1959 (26)	383.1965	+1.6	$C_{22}H_{27}N_2O_4^+$	**A**
351.1697 (11)	351.1703	+1.7	$C_{21}H_{23}N_2O_3^+$	**B** (**A**–CH_3OH)
295.1070 (14)	295.1077	+2.4	$C_{17}H_{15}N_2O_3^+$	**C** (**B**–C_4H_8)
255.1487 (13)	255.1492	+2.0	$C_{16}H_{19}N_2O^+$	**D** (**A**–$C_6H_8O_3$)
201.1016 (20)	201.1022	+3.0	$C_{12}H_{13}N_2O^+$	**E** (**D**–C_4H_6)
174.0909 (100)	174.0913	+2.3	$C_{11}H_{12}NO^+$	**F** (**E**–HCN)
156.0803 (18)	156.0808	+3.2	$C_{11}H_{10}N^+$	**G** (**F**–H_2O)
146.0596 (20)	146.0600	+2.7	$C_9H_8NO^+$	**H** (**E**–C_2H_4–HCN)
**3c**				
387.1711 (20)	387.1715	+1.0	$C_{21}H_{24}FN_2O_4^+$	**A**
355.1447 (13)	355.1452	+1.4	$C_{20}H_{20}FN_2O_3^+$	**B** (**A**–CH_3OH)
299.0819 (14)	299.0826	+2.3	$C_{16}H_{12}FN_2O_3^+$	**C** (**B**–C_4H_8)
259.1236 (15)	259.1241	+1.9	$C_{15}H_{16}FN_2O^+$	**D** (**A**–$C_6H_8O_3$)
205.0768 (41)	205.0772	+2.0	$C_{11}H_{10}FN_2O^+$	**E** (**D**–C_4H_6)
178.0659 (100)	178.0663	+2.2	$C_{10}H_9FNO^+$	**F** (**E**–HCN)
160.0553 (26)	160.0557	+2.5	$C_{10}H_7FN^+$	**G** (**F**–H_2O)
150.0345 (38)	150.0350	+3.3	$C_8H_5FNO^+$	**H** (**E**–C_2H_4–HCN)

[a]Relative intensities are provided in parentheses.
[b]Only ions with relative intensities higher than 5% are reported.

Table S4. ^{13}C NMR data of mitraphylline (**1**) isolated from *U. guianensis* shoots after incorporation of 1-^{13}C-D-glucose into cultures (DMSO-d_6).

			Relative intensity of signal		
			**L**	**U**	**ΔC**
**C**	δ_c	δ_c*			
**2**	180.5	180.9	1.5	1.4	1.18
**3**	74.1	74.6	1.8	2.3	0
**5**	53.3	54.3	2.1	1.9	1.21
**6**	34.7	35.1	2.8	1.9	**1.62**
**7**	55.4	55.5	2. 0	2.6	0
**8**	134.0	133.3	0.9	0.9	0
**9**	123.6	123.0	2.5	1.3	**2.11**
**10**	122.3	122.6	1.1	1.3	0
**11**	128.2	128.0	1.7	1.5	1.24
**12**	109.5	109.5	1.5	1.8	0
**13**	142.1	140.6	1.3	0.6	**2.38**
**14**	28.4	28.3	2.1	1.6	**1.44**
**15**	30.1	30.4	1.6	2.4	0
**16**	106.9	106.9	1.0	1.6	0
**17**	154.2	154.0	2.1	1.6	**1.44**
**18**	15.1	14.8	5.1	3.4	**1.65**
**19**	73.8	73.8	1.9	2.4	0
**20**	40.6	40.5	1.7	2.1	0
**21**	53.8	54.3	2.0	1.7	**1.30**
**22**	166.7	167.1	1.5	1.9	0
**23-OCH$_3$**	51.1	50.7	5.3	3.3	**1.77**
*** literature[1]**					

U: control experiments with unlabeled precursor; L: labeling experiment with ^{13}C precursor; ΔC= 1.1% x L/U: increase in the relative intensity (significant increases in bold for enriched carbons).

Table S5. ^{13}C NMR data of isomitraphylline (**3**) isolated from *U. guianensis* shoots after incorporation of 1-^{13}C-D-glucose into cultures (DMSO-d_6).

			Relative intensity of signal		
			**L**	**U**	**ΔC**
**C**	**δ_c**	**δ_c***			
**2**	179.9	181.6	1.6	1.5	1.17
**3**	71.1	71.6	2.1	2.2	0
**5**	52.7	53.3	2.2	1.9	1.27
**6**	34.8	35.3	3.9	1.9	**2.26**
**7**	55.7	56.4	2.4	2.2	1.20
**8**	133.6	133.7	0.8	1.2	0
**9**	124.3	124.7	2.9	1.5	**2.13**
**10**	121.5	122.2	1.4	1.6	0
**11**	127.6	127.4	1.9	1.5	1.39
**12**	109.3	109.6	1.9	1.7	1.22
**13**	141.5	140.3	1.7	1.1	**1.70**
**14**	28.7	29.1	2.6	1.7	**1.68**
**15**	29.4	29.9	2.0	2.1	1.05
**16**	106.7	107.2	1.2	1.5	0
**17**	153.7	153.7	2.2	1.5	**1.61**
**18**	14.7	14.8	5.7	3.2	**1.96**
**19**	73.3	73.8	2.4	2.4	0
**20**	40.6	40.8	2.0	2.2	0
**21**	53.4	54.2	2.7	1.6	**1.86**
**22**	166.1	167.0	1.5	1.5	0
**23-OCH_3**	50.7	50.7	5.4	3.2	**1.86**
*** literature[1]**					

U: control experiments with unlabeled precursor; L: labeling experiment with ^{13}C precursor; ΔC= 1.1% x L/U: increase in the relative intensity (significant increases in bold for enriched carbons).

Table S6. ^{13}C NMR data of isorhynchophylline (**4**) isolated from *U. guianensis* shoots after incorporation of 1-^{13}C-D-glucose into cultures (DMSO-d_6).

			**Relative intensity of signal**		
			**L**	**U**	**ΔC**
**C**	**δ_c**	**δ_c***			
**2**	180.2	182,4	2.5	2.3	1.19
**3**	72.0	72.2	1.0	1.0	0
**5**	53.5	54.2	4.5	4.5	0
**6**	34.7	36.5	2.0	0.9	**2.4**
**7**	56.0	57.0	4.5	6,4	0
**8**	133.8	134.2	0.9	1.1	0
**9**	124.4	125.2	5.8	3.9	**1.6**
**10**	121.4	122.1	0.7	0.7	0
**11**	127.4	127.1	4.0	4.0	0
**12**	109.0	109.6	3.5	4.2	0
**13**	141.3	140.7	2.0	2.0	0
**14**	30.0	30.1	1.1	0.5	**2.4**
**15**	37.3	38.3	1.7	1.7	0
**16**	110.8	113.0	1.0	1.1	0
**17**	160.2	159.5	1.0	0.7	**1.6**
**18**	11.0	11.2	2.2	1.5	**1.6**
**19**	23.8	24.3	3.4	4.1	0
**20**	37.3	38.3	1.7	1.7	0
**21**	57.7	58.2	1.7	1.1	**1.7**
**22**	166.6	168.4	0,3	0.3	0
**23-OCH_3**	50.4	50.9	1.6	0.7	**2.5**
**24- OCH_3**	61.3	61.2	1.7	0.7	**2.7**
*** literature[1]**					

U: control experiments with unlabeled precursor; L: labeling experiment with ^{13}C precursor; ΔC= 1.1% x L/U: increase in the relative intensity (significant increases in bold for enriched carbons).

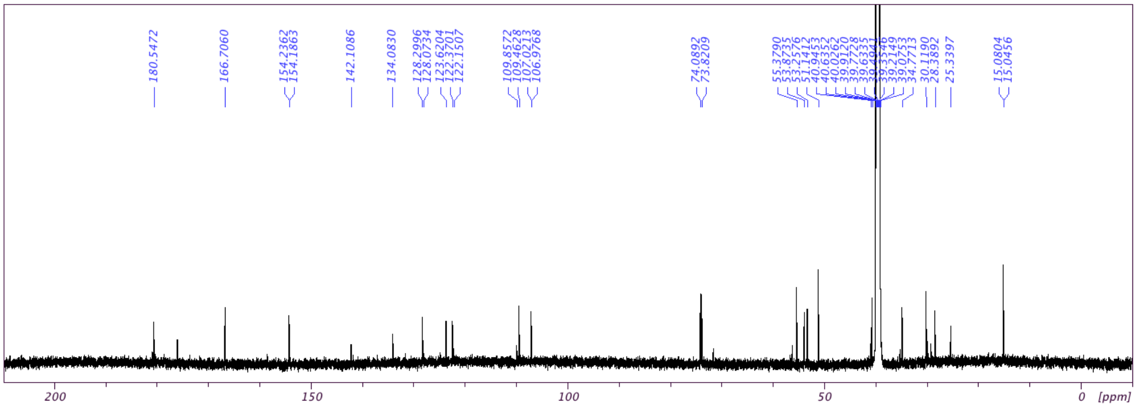

Figure S38. ^{13}C NMR (150 MHz, DMSO-d$_6$) spectrum of **1** with natural ^{13}C-isotopic abundance.

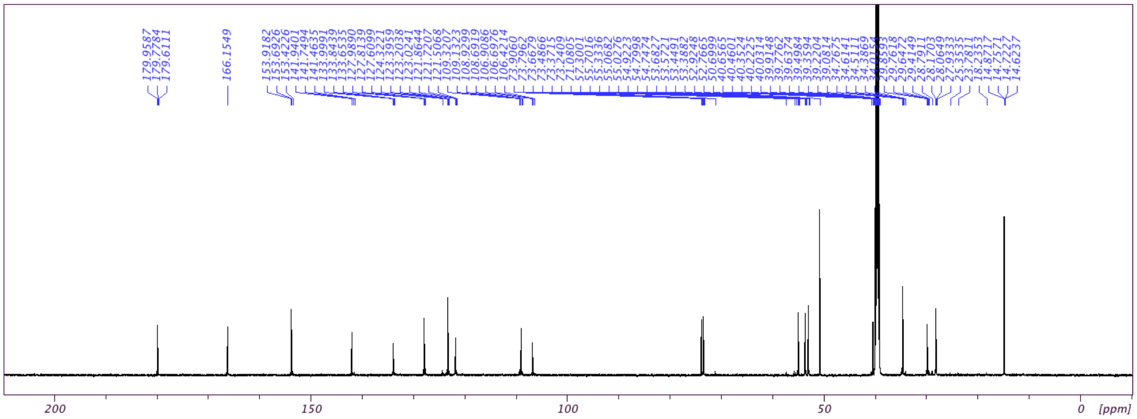

Figure S39. ^{13}C NMR (150 MHz, DMSO-d$_6$) spectrum of **1** after *U. guianensis* plantlets grew on culture with 1-^{13}C-D-glucose.

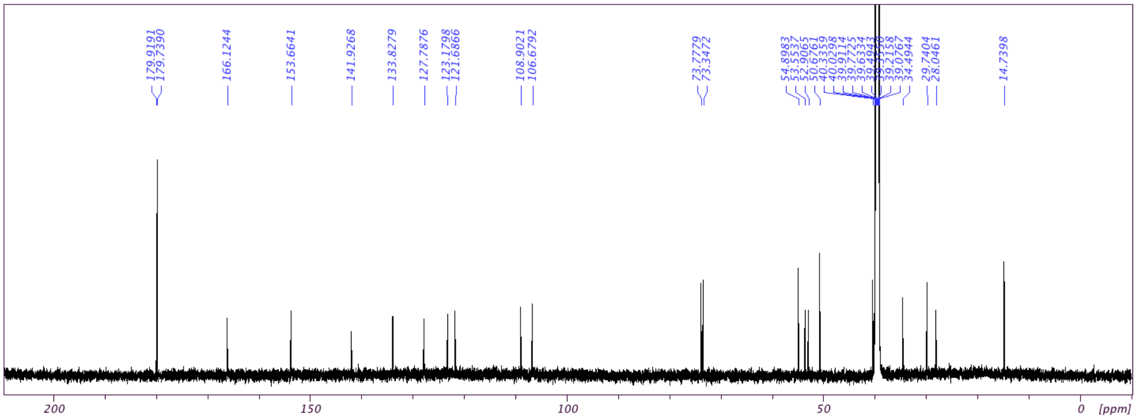

Figure S40. ^{13}C NMR (150 MHz, DMSO-d6) spectrum of **1** after *U. guianensis* plantlets grew on culture with 2-^{13}C-tryptophan.

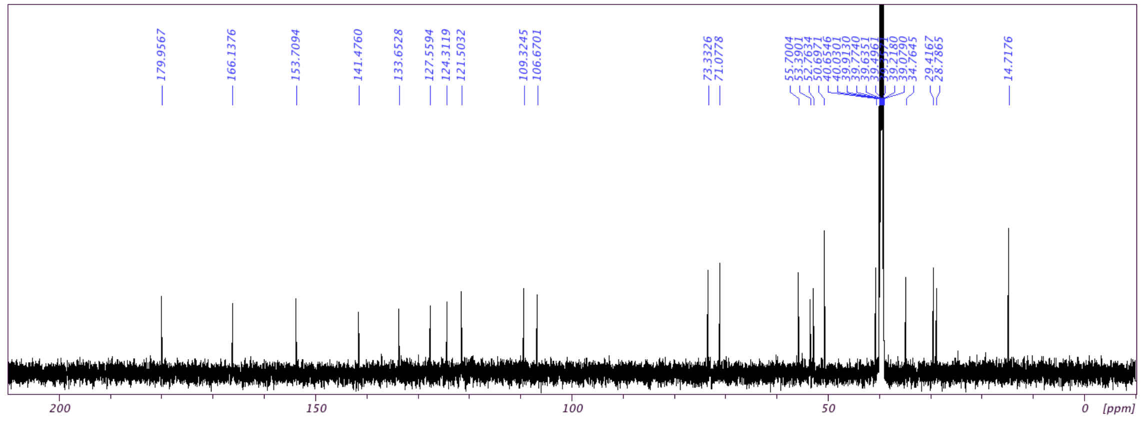

Figure S41. ^{13}C NMR (150 MHz, DMSO-d$_6$) spectrum of **3** with natural ^{13}C-isotopic abundance.

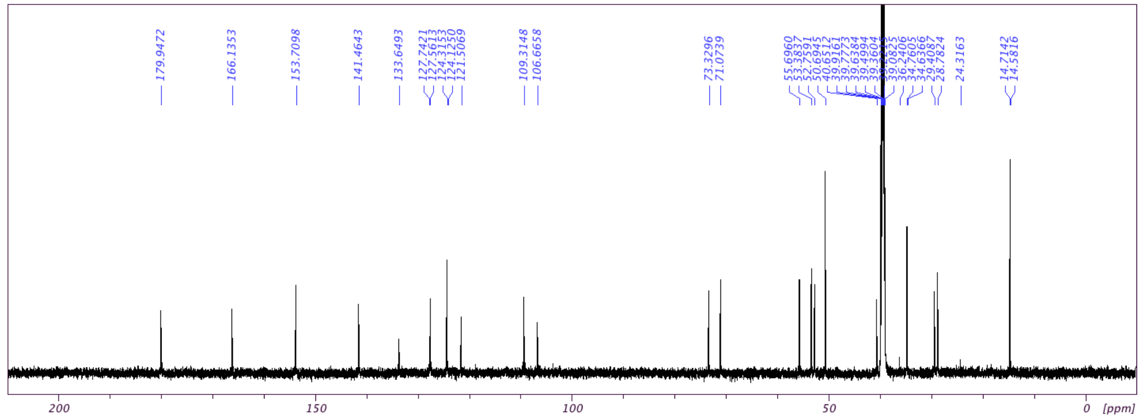

Figure S42. ^{13}C NMR (150 MHz, DMSO-d$_6$) spectrum of **3** after *U. guianensis* plantlets grew on culture with 1-^{13}C-D-glucose.

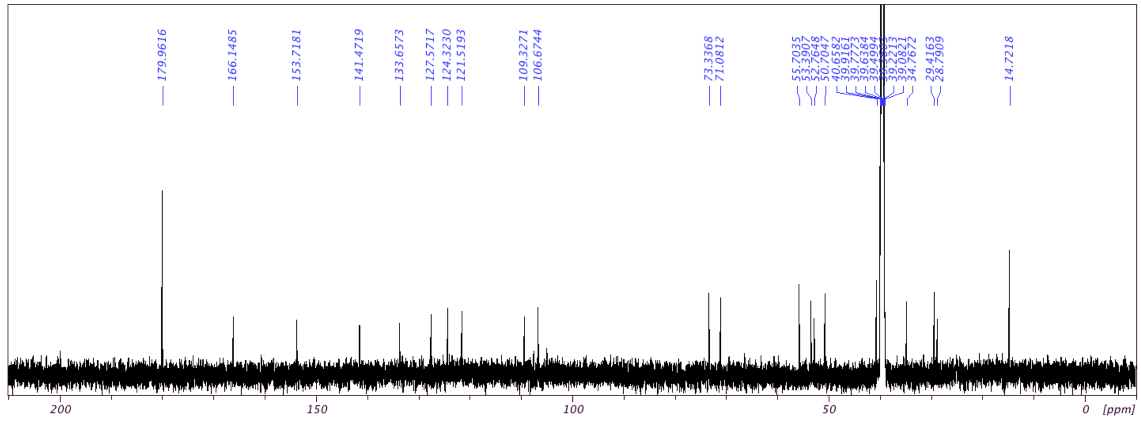

Figure S43. ^{13}C NMR (150 MHz, DMSO-d6) spectrum of **3** after *U. guianensis* plantlets grew on culture with 2-^{13}C-tryptophan.

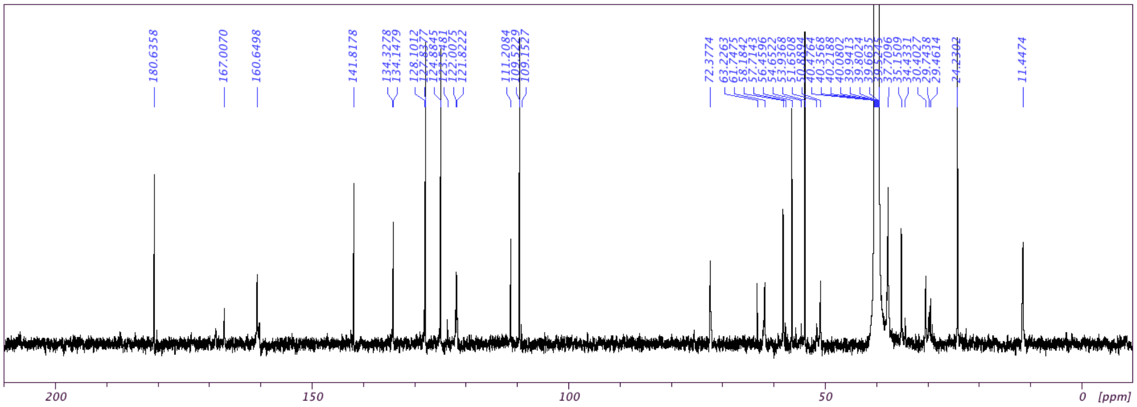

Figure S44. ^{13}C NMR (150 MHz, DMSO-d$_6$) spectrum of **4** with natural ^{13}C-isotopic abundance.

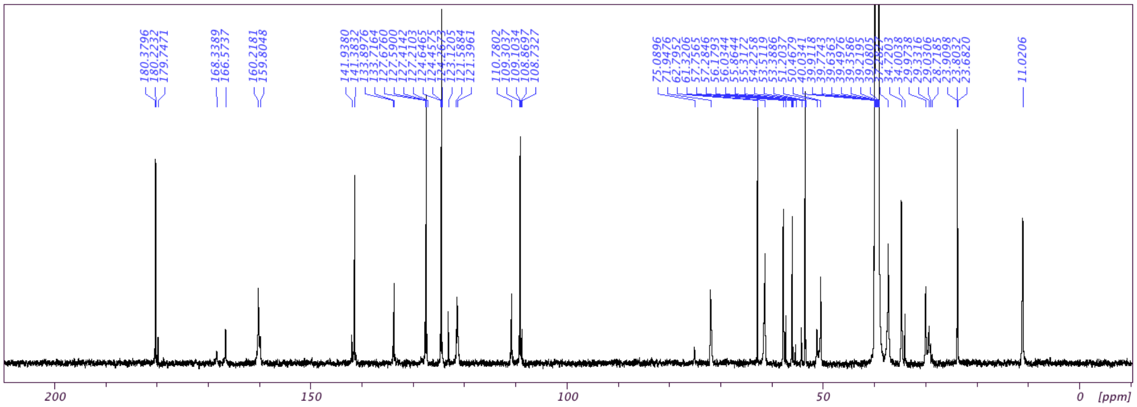

Figure S45. ^{13}C NMR (150 MHz, DMSO-d$_6$) spectrum of **4** after *U. guianensis* plantlets grew on culture with 1-^{13}C-D-glucose.

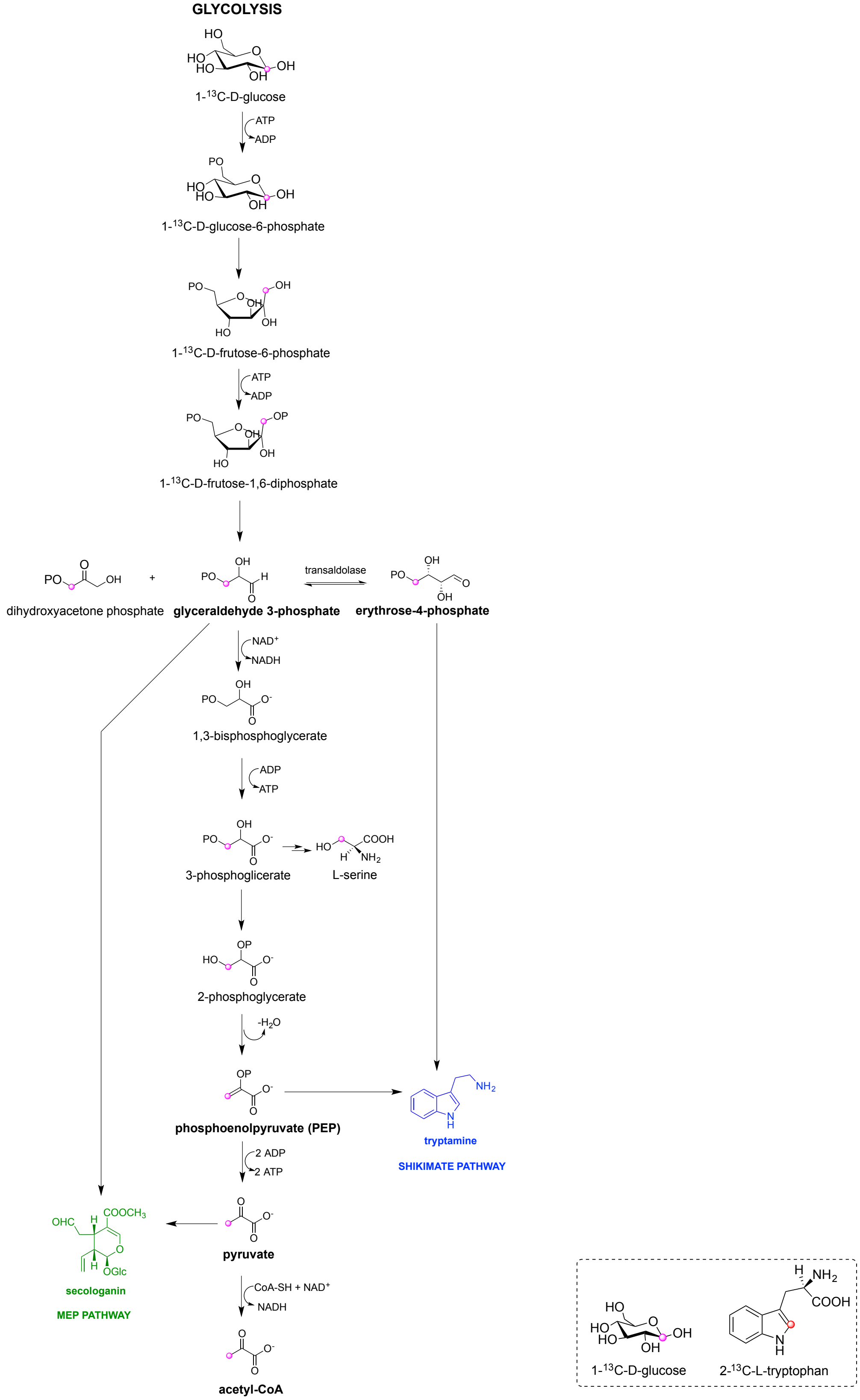

Figure S46. Biosynthetic pathway to oxindole alkaloids scaffolds (**1-4**) derived from 1-^{13}C-D-glucose and 2-^{13}C-tryptophan.

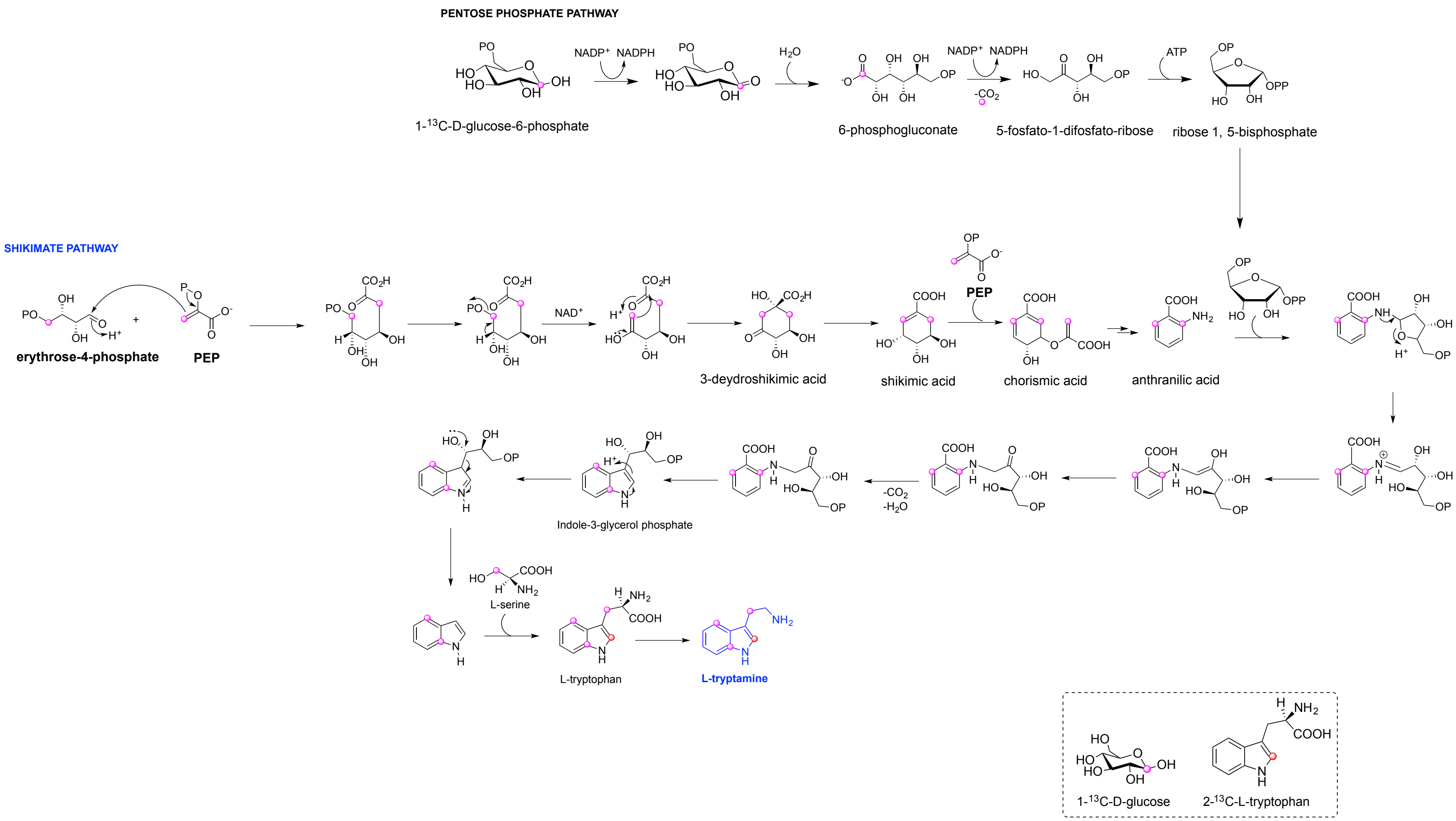

Figure S46 (continued)

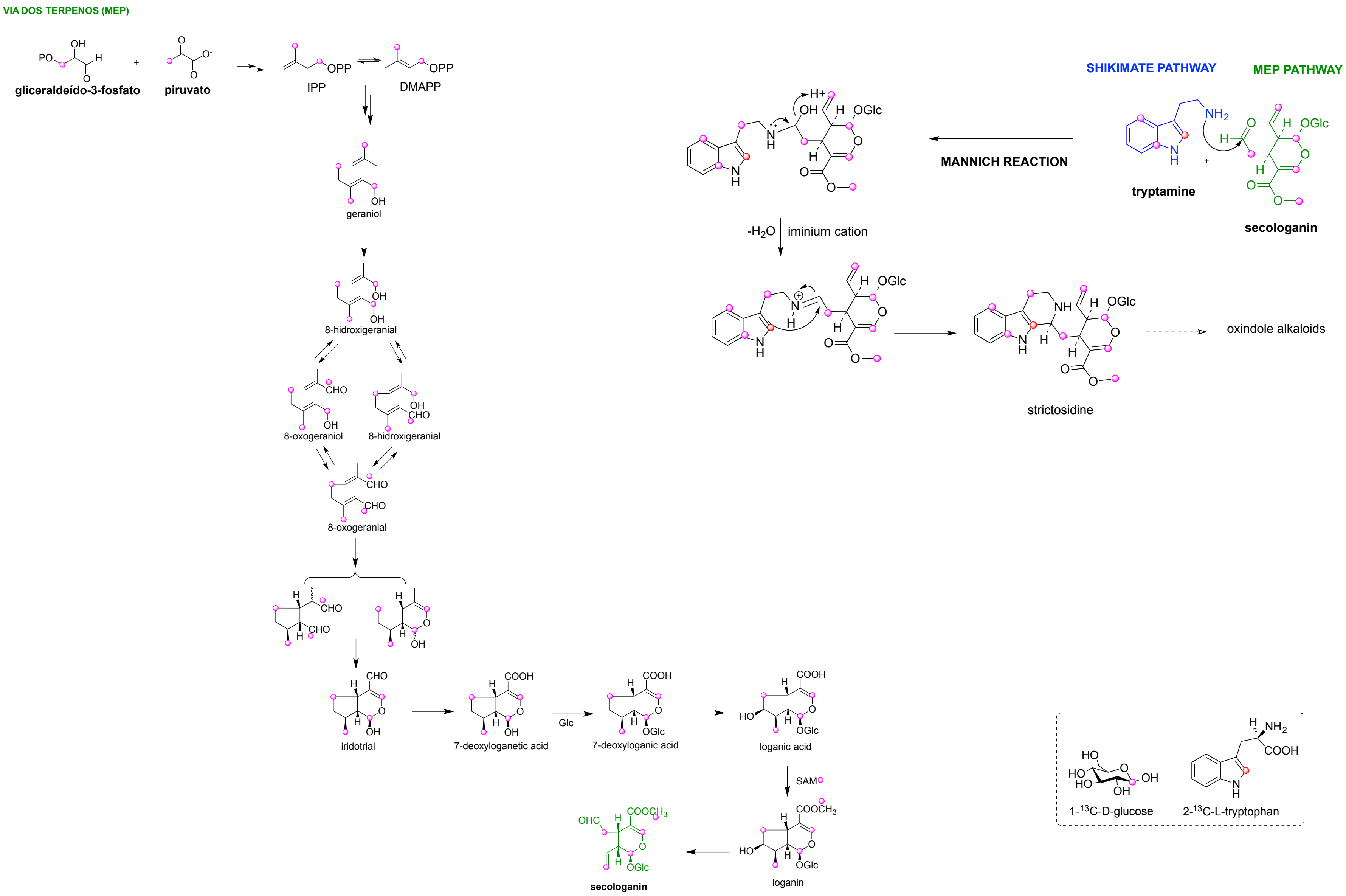

Figure S46 (continued)

Reference

1) a) Toure, H., Babadjamian, A., Balansard, G., Faure, R., Houghtons, P. J. Complete ^{1}H and ^{13}C NMR chemical shift assignments for some pentacyclic oxindole alkaloids. *Spectrosc. Lett.*, **25**, 293-300 (1992); b) Seki, H., Takayama, H., Aimi, N., Sakal, S., Ponglux, D. A nuclear magnetic resonance study on the eleven stereoisomers of heteroyohimbine-type oxindole alkaloid. *Chem. Pharm. Bull.* **41**, 12, 2077-2086 (1993); c) Carbonezi, C. A., Hamrski, L. H., Junior, O. A. F., Furlan, M., Bolzani, V. Da S., Young, M. C. M. Determinação por RMN das configurações relativas e conformações de alcalóides oxindólicos isolados de *Uncaria guianensis*. *Quim. Nova* **27**, 6, 878-881 (2004).
